# Antioxidant and Anti‐Senescence Polyvinyl Alcohol‐Gallic Acid Supramolecular Hydrogels for Stem Cell Culture

**DOI:** 10.1002/adhm.202402882

**Published:** 2025-04-17

**Authors:** Yiduo Zhou, Matías L. Picchio, Yan Nie, Lei Wang, Oihane Sanz, Yue Liu, Xun Xu, Lukas Prantl, Oliver Felthaus, Weiwei Wang, Marcelo Calderón, Nan Ma

**Affiliations:** ^1^ Institute of Chemistry and Biochemistry Free University of Berlin 14195 Berlin Germany; ^2^ Institute of Active Polymers Helmholtz‐Zentrum Hereon 14513 Teltow Germany; ^3^ Institute of Functional Materials for Sustainability Helmholtz‐Zentrum Hereon 14513 Teltow Germany; ^4^ POLYMAT, Applied Chemistry Department, Faculty of Chemistry University of the Basque Country UPV/EHU Paseo Manuel de Lardizábal 3 20018 Donostia‐San Sebastián Spain; ^5^ IKERBASQUE Basque Foundation for Science Plaza Euskadi 5 Bilbao 48009 Spain; ^6^ Universidad Tecnológica Nacional Facultad Regional Villa María Av. Universidad 450 (5900) Villa María Córdoba Argentina; ^7^ Consejo Nacional de Investigaciones Científicas y Técnicas (CONICET) Godoy Cruz 2290, CABA Buenos Aires Argentina; ^8^ Department of Plastic, Hand and Reconstructive Surgery University Hospital Regensburg Franz‐Josef‐Strauß‐Allee 11 93053 Regensburg Germany

**Keywords:** cell senescence, gallic acid, mesenchymal stem cells, mitochondria, reactive oxygen species (ROS)

## Abstract

Replicative senescence presents a significant challenge in mesenchymal stem cell (MSC) expansion due to high reactive oxygen species (ROS) levels generated during culture. Elevated ROS levels lead to oxidative stress, cellular damage, and senescence, limiting the biomedical applications of MSCs. In this study, a supramolecular thermo‐reversible hydrogel composed of the natural polyphenolic compound gallic acid (GA) and polyvinyl alcohol (PVA) was designed to scavenge ROS and mitigate MSC senescence. The PVA‐GA hydrogel, stabilized by strong hydrogen bonding forces, exhibited an elastic modulus comparable to that of human soft tissue and facilitated the sustained release of GA over 14 days. It enhanced MSC survival, protected against oxidative stress, reduced intracellular ROS levels, diminished mitochondrial damage, and decreased cellular senescence. The hydrogel maintained the multilineage differentiation potential and typical phenotype of MSCs. Additionally, it preserved vascular endothelial growth factor (VEGF) secretion from MSCs under oxidative stress and enhanced their pro‐angiogenic effect. The conditioned medium derived from MSCs in the hydrogel group promoted migration and tube formation of human umbilical vein endothelial cells (HUVECs). These findings suggest that the PVA‐GA hydrogel holds significant promise for the biomedical applications of MSCs, potentially addressing the challenges posed by oxidative stress and cellular senescence.

## Graphical abstract

The PVA‐GA hydrogel is formed through hydrogen bonding between PVA hydroxyl groups and GA galloyl units, enabling sustainable GA release and its extended biological activity. The hydrogel scavenges ROS to reduce oxidative stress, preventing mitochondrial damage from excessive ROS, mitigating hADSC senescence, and preserving VEGF secretion, offering a potential solution for biomedical applications.

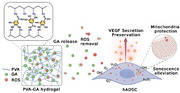

## 1 Introduction

Mesenchymal stem cells (MSCs) are widely recognized for their therapeutic potential in treating various human diseases. The versatility and multipotency of MSCs, along with their capacity to modulate immune responses and secrete bioactive factors, make them powerful tools for a wide range of clinical applications.^[^ ^1^, ^2^ ^]^ As of our search date (July 1, 2024), nearly 1500 clinical trials involving MSCs have been registered on ClinicalTrials.gov using “mesenchymal stem cells” as the Intervention/Treatment keywords. This reflects a significant increase over the past three decades since the first report in 1995.^[^ ^3^ ^]^ Ongoing research continues to expand the therapeutic applications of MSCs, establishing them as a cornerstone of advanced therapeutic strategies.^[^ ^4^, ^5^ ^]^ However, to achieve a sufficient cell number for therapeutic use, MSCs must be expanded through in vitro culture. Replicative senescence, a naturally occurring event during the culture process, leads to reduced cell proliferation and compromised cellular functions, which poses significant challenges for their therapeutic applications.^[^ ^6^, ^7^, ^8^ ^]^ Key features of replicative senescence include irreversible cell cycle arrest, telomere shortening, chromatin remodeling, and the secretion of pro‐inflammatory cytokines and other components of the senescence‐associated secretory phenotype (SASP), such as increased senescence‐associated β‐galactosidase (SA‐β‐Gal) activity. These characteristics contribute to decreased therapeutic efficacy.^[^ ^9^ ^]^ Reactive oxygen species (ROS) play a crucial role in the process of replicative senescence. ROS can cause damage to various cellular biomolecules, including DNA, proteins, and lipids.^[^ ^10^ ^]^ Additionally, ROS can harm mitochondrial components and activate senescence pathways, such as the p53/p21 and p16^INK4a^ signaling pathways.^[^ ^11^ ^]^ Such oxidative stress negatively impacts the function of MSCs, leading to increased cell senescence, apoptosis, mitochondrial dysfunction, and altered differentiation potential.^[^ ^12^, ^13^, ^14^, ^15^ ^]^ Therefore, sustainably mitigating excess ROS in the MSC microenvironment could be an effective strategy to enhance their therapeutic potential.

Various strategies have been developed to scavenge excess ROS from the stem cell microenvironment. Our previous work demonstrated the use of polydopamine (PDA) for the removal of external ROS, which successfully preserved MSCs’ stemness and mitigated senescence.^[^ ^12^ ^]^ Recently, naturally derived compounds, especially polyphenols, have shown significant potential in mitigating the aging process of cells.^[^ ^16^, ^17^, ^18^ ^]^ Among these, gallic acid (GA) has been extensively studied and is recognized for its broad spectrum of biological activities, including its antioxidant properties. GA effectively neutralizes free radicals, thereby protecting cells from oxidative damage.^[^ ^19^ ^]^ Moreover, GA exhibits anti‐inflammatory, anticancer, and antimicrobial properties, making it a versatile compound for therapeutic applications.^[^ ^20^, ^21^ ^]^ In the context of stem cell research, GA has been identified as a geroprotector, demonstrating senescence‐attenuating effects on human MSCs.^[^ ^22^ ^]^ Hydrogels incorporating GA as a functional component can effectively protect stem cells from oxidative stress, thereby enhancing their proliferation, pro‐angiogenic capacity, modulation of inflammation, and differentiation potential.^[^ ^23^, ^24^, ^25^ ^]^ However, it is critical to note that GA can exhibit dual characteristics, functioning as both an antioxidant and a pro‐oxidant.^[^ ^19^, ^26^ ^]^ Its pro‐oxidant properties are primarily associated with the induction of apoptosis by generating ROS that trigger programmed cell death. In contrast, its antioxidant activity and free radical scavenging ability play a protective role by safeguarding biological cells, tissues, and organs from oxidative stress‐induced damage. Since GA's antioxidant or pro‐oxidant behavior is influenced by its concentration and the ROS levels in the surrounding environment,^[^ ^19^, ^21^ ^]^ the development of a precise and controlled release system for GA is essential to protect cells from oxidative stress and maximize its therapeutic potential.

Recently, a novel supramolecular hydrogel composed of polyvinyl alcohol (PVA) and GA has drawn substantial attention for diverse biomedical applications such as wound healing, tissue engineering, topical drug delivery, and 3D‐bioprinting.^[^ ^27^, ^28^, ^29^, ^30^ ^]^ PVA, a widely used synthetic polymer known for its excellent biocompatibility, chemical stability, and mechanical properties, was used as a matrix material in such hydrogels, while GA was applied as a crosslinker. Structurally, the interaction between GA and PVA is primarily driven by hydrogen bonds between the phenolic hydroxyl groups in GA and the hydroxyl groups in PVA. Additionally, π‐π stacking and hydrophobic interactions may also contribute to the formation and stability of the hydrogel.^[^ ^27^ ^]^ The hydrogen bonding between GA and PVA drives both the formation and disassembly of the hydrogel, which can be leveraged to achieve the sustained release of GA. Such interactions also affect the properties of the hydrogel, including gelation kinetics, phase transition temperatures, and viscoelasticity. By fine‐tuning the proportions of GA, the hydrogel can be endowed with distinct biological functions and mechanical properties.

**Figure 1:**
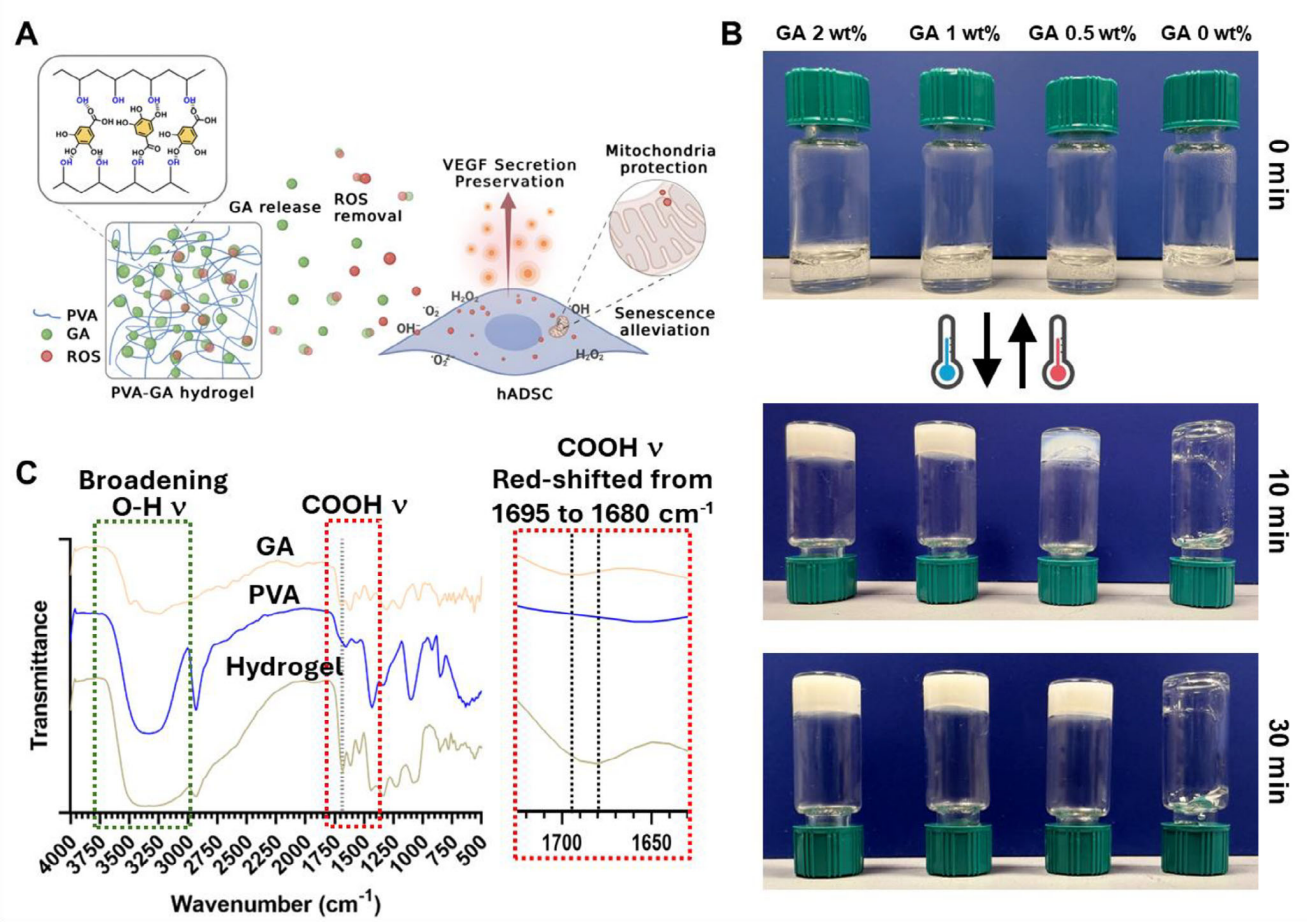
Preparation of PVA‐GA hydrogels as a ROS scavenger. A) PVA‐GA hydrogel is formed through hydrogen bonding between the hydroxyl groups of PVA and the galloyl units of GA. GA is sustainably released from the hydrogel. Both the released and encapsulated GA react with ROS to reduce oxidative stress in the cell culture environment, preventing mitochondrial damage caused by excessive ROS, mitigating hADSC senescence, and preserving VEGF secretion. B) Thermo‐reversible gel‐sol phase transition of PVA‐GA hydrogels with a fixed PVA concentration (10 wt.%) and varied GA concentrations. C) FTIR spectra of PVA‐GA hydrogel (GA 1 wt.%) and the pure components. The peak at 1695 cm^−1^, corresponding to ‐COOH stretching vibration in GA, is red‐shifted to 1680 cm^−1^ in the PVA‐GA hydrogel.

In this study, supramolecular PVA‐GA hydrogels were prepared based on the hydrogen bonding between the hydroxyl groups of PVA and the galloyl units of GA (**Figure** **1**A). The release of GA and its effect on human adipose‐derived stem cells (hADSCs) were investigated. Our results demonstrate that the PVA‐GA hydrogel effectively reduced oxidative stress in hADSCs, maintained mitochondrial integrity, preserved the secretion of vascular endothelial growth factor (VEGF) and the multipotency of MSC differentiation under elevated oxidative stress, while also mitigating cellular senescence. These findings highlight the potential of PVA‐GA hydrogels, in combination with stem cells, for biomedical applications.

## 2 Results and Discussion

### 2.1 PVA‐GA Hydrogel Formation Through Hydrogen Bonding

Small phenolic molecules, such as GA, have been demonstrated to strongly bind to PVA in water, triggering the formation of elastic hydrogels.^[^ ^29^ ^]^ As illustrated in Figure 1A, robust hydrogen bonding between the hydroxyl groups of PVA and galloyl units of GA renders highly stable networks. The prepared hydrogel could reversibly transition from a gel to a liquid state, with controllable gelation dynamics via modulating the PVA:GA ratio in the hydrogel (Figure 1B). As the GA concentration decreased, the gelation kinetics slowed. Pure PVA could not form a stable gel at 4 °C for 30 min. Gelation was rapidly achieved in 10 min for hydrogels with GA concentrations of 2 wt.% and 1 wt.%. However, for the hydrogel with a GA concentration of 0.5 wt.%, the gelation process was prolonged to 30 min. Fourier‐transform infrared (FTIR) analysis revealed significant changes in the characteristic vibrational modes of PVA and GA after hydrogel formation (Figure 1C). Specifically, the band centered ≈3300 cm^−1^ attributed to ‐OH stretching in pure GA and PVA showed significant broadening, while the peak at 1695 cm^−1^ corresponding to ‐COOH stretching in GA red‐shifted to 1680 cm^−1^ in the PVA‐GA hydrogel. These results provide evidence that hydrogen bonding is likely the primary driving force for the hydrogel assembly.^[^ ^29^ ^]^

### 2.2 Physicochemical Properties of PVA‐GA Hydrogel

**Figure 2:**
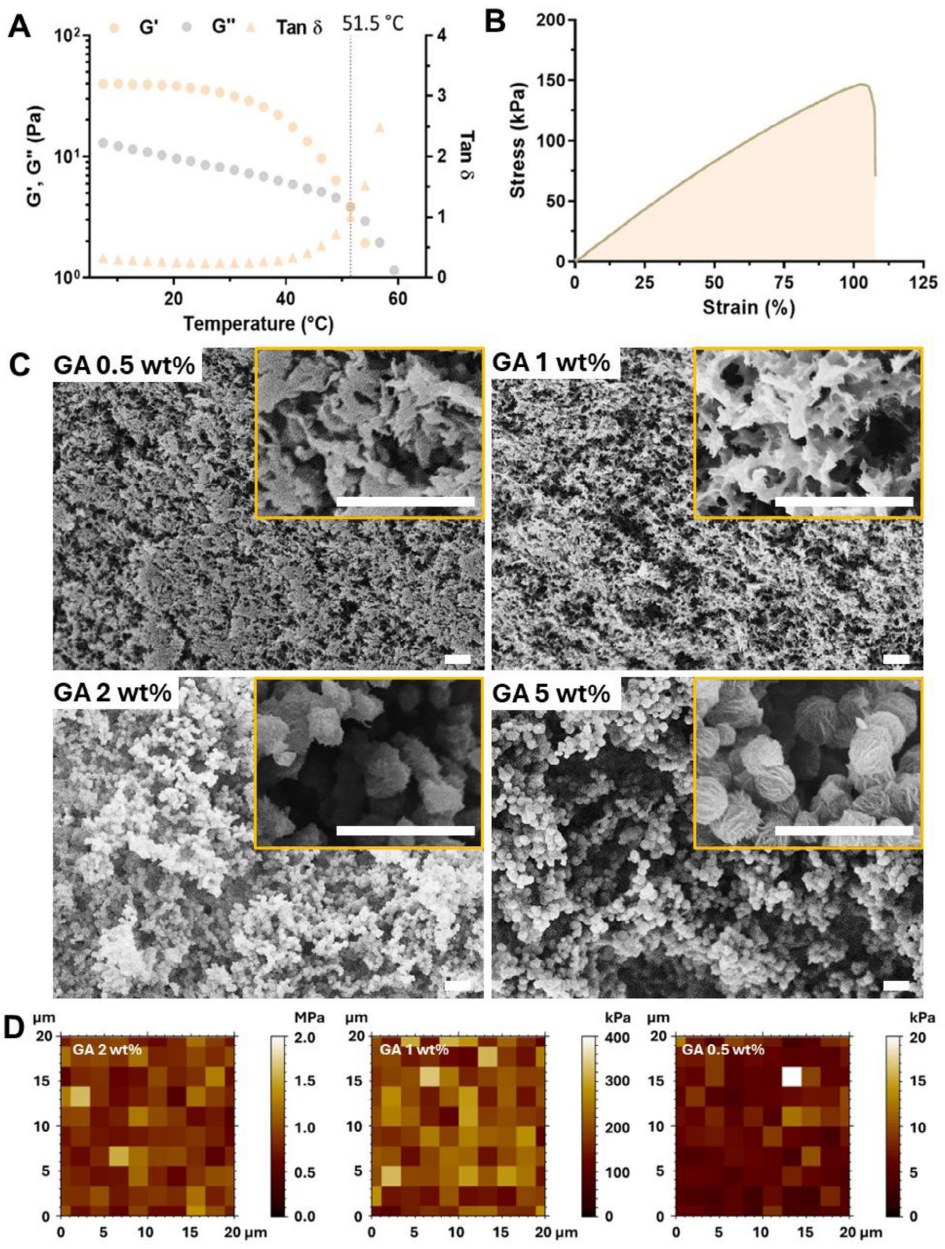
Mechanical properties and nano‐morphology of PVA‐GA hydrogels. A) Temperature sweep for the PVA‐GA hydrogel (GA 1 wt.%). B) Stress versus strain curve of the PVA‐GA hydrogel (GA 1 wt.%). C) Scanning electron microscopy (SEM) images showing the internal morphology of the PVA‐GA hydrogels with varying GA concentrations (0.5, 1, 2, and 5 wt.%); scale bar = 10 µm. D) Young's modulus maps of the hydrogel surface obtained through AFM nanoindentation.

The dynamic hydrogen bonds in the PVA‐GA hydrogel enable a low gel‐to‐sol phase transition temperature (T_gel‐sol_) of ≈52 °C, as determined by the cross‐over point of the elastic (G') and viscous (G″) moduli in dynamic mechanical thermal experiments (**Figure** **2**A). We then investigated the viscoelastic properties of the PVA‐GA hydrogel using small amplitude oscillatory shear (SAOS). Amplitude sweeps showed a linear viscoelastic range (LVR) of 3% for this gel (Figure S1A, Supporting Information), while frequency sweeps revealed that G′ was higher than G″ across the typical range of 0.1–100 rad s^−1^ without a cross‐over point, resembling the behavior of covalent‐cross‐linked hydrogels (Figure S1B, Supporting Information). Notably, this is a significant advantage of these hydrogels. While the supramolecular interactions in PVA‐GA systems effectively function like covalent bonds, the hydrogels retain their ability to undergo thermal processing due to their low T_gel‐sol_.

The strong binding between PVA and GA is also demonstrated in the excellent mechanical performance of the hydrogel, exhibiting a stretchability of over 100%, a tensile strength of ≈150 kPa (Figure 2B), and a toughness of 90 kJ m^−^^3^. Additionally, the Young's modulus of this material was 167 kPa, which is comparable to the stiffness of some biological tissues such as the skin and certain regions of the heart (100–300 kPa).^[^ ^31^ ^]^ The stress‐relaxation behavior of the hydrogel was also investigated to provide insights into the connectivity and chain mobility within the network (Figure S1C, Supporting Information). The hydrogel reached equilibrium stress at ≈60% of the instantaneous values, which is higher than that typically observed for supramolecular hydrogels,^[^ ^32^ ^]^ further supporting the unusually robust interaction between PVA and GA. It is noteworthy that the relaxation time in this network exceeds 600 s, resulting in materials with poor chain mobility, likely limiting the adhesion of the hydrogel. In fact, the hydrogel's maximum adhesive stress and adhesion energy were 730 Pa and 256 mJ m^−^^2^ (Figure S1D, Supporting Information), significantly lower than those reported for PVA hydrogels using borax as a dynamic crosslinker.^[^ ^33^ ^]^ Adhesion could be improved by decreasing the GA amount in the gel formulation, but concentrations lower than the selected 1 wt.% result in slow gelation kinetics (Figure 1B). When the hydrogel is applied for cell encapsulation, such slow gelation kinetics may lead to significant cell sedimentation during the gelation process (Figure S2, Supporting Information). In addition, a prolonged period of incubation at 4 °C may induce cell death.^[^ ^34^ ^]^

Conversely, increasing the polyphenol content can accelerate gelation without compromising adhesion performance. However, the GA concentration significantly impacts the microstructural arrangement of these hydrogels and their mechanical properties. While 1 wt.% of GA yields well‐interconnected, low‐porosity polymer networks with good elasticity, increasing concentrations up to 5 wt.% result in mechanically weak materials with a highly porous and globular internal morphology (Figure 2C). Textural properties measured by N_2_ physisorption revealed that the pore volume (V_p_) of the hydrogels increased from 0.016 to 0.067 cm^3^ g^−1^ for 1 wt.% and 5 wt.% of GA respectively (Figure S3, Supporting Information). Increasing GA also stiffened the material, as confirmed by atomic force microscopy (AFM) indentation measurements of the hydrogel surface. The Young's modulus, as measured by nanoindentation, increased from 6.3 ± 2.4 to 107 ± 26 kPa as the GA concentration increased from 0.5 wt.% to 1 wt.% and reached 867 ± 216 kPa for the hydrogel with 2 wt.% GA (Figure 2D). These results suggest that by adjusting the PVA:GA ratio, the microstructures and mechanical properties of the hydrogels could be precisely tuned, thereby expanding their potential for a wide range of biomedical applications.

### 2.3 Release of Gallic Acid from Hydrogel

**Figure 3:**
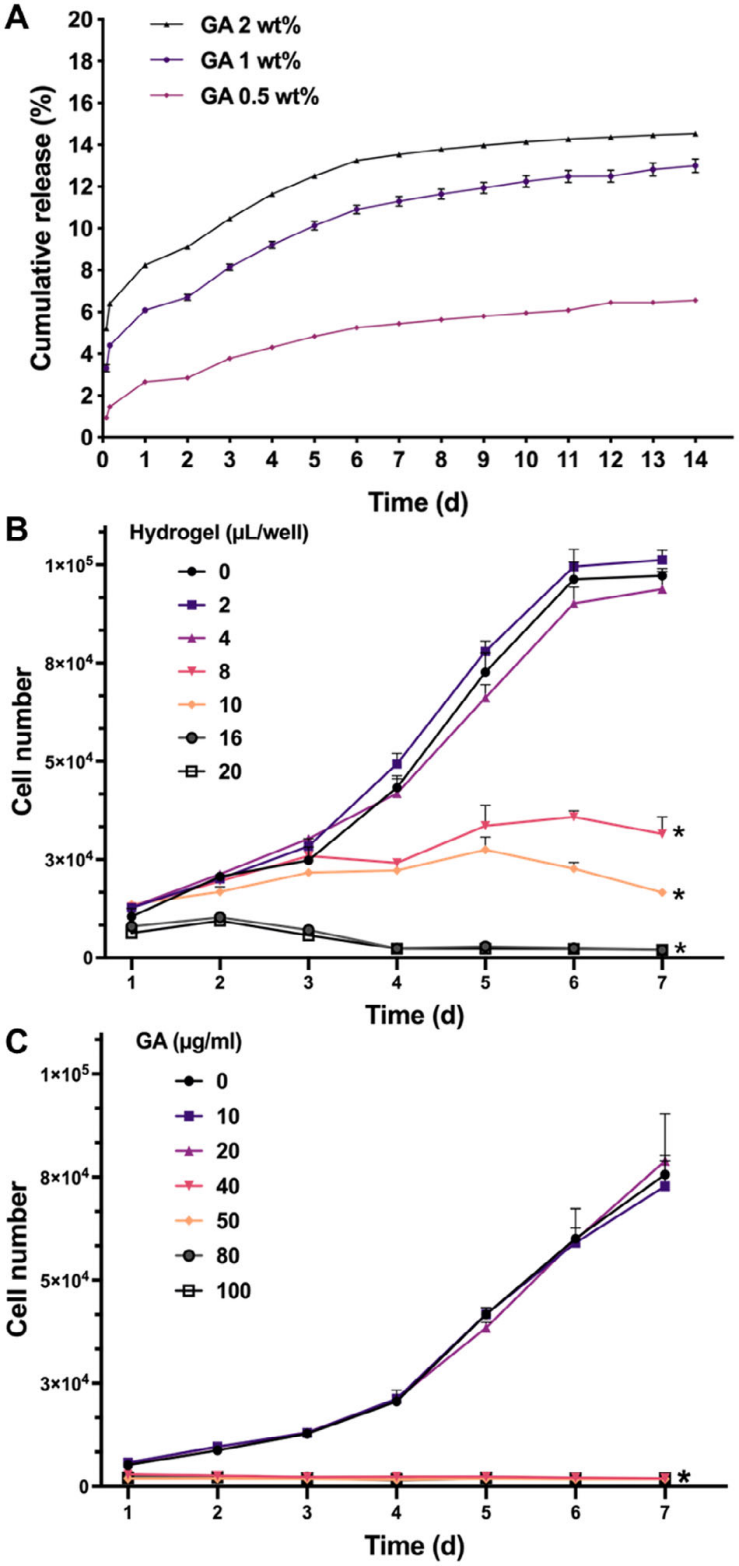
Release of GA from hydrogel and the effect of hydrogel dosage and GA concentration on hADSC proliferation. A) Cumulative release of GA from hydrogels with varying GA concentrations in distilled water (*n* = 3). B) Growth curves of cells in the presence of hydrogel (GA 1 wt.%) at different dosages (*n* = 3, *p < 0.05 compared to the group without hydrogel, two‐way ANOVA with Tukey's multiple comparisons test). C) Growth curves of cells treated with different concentrations of GA. The GA concentration in each group corresponds to that in the hydrogel samples presented in (B) (*n* = 3, *p < 0.05 compared to the group without GA, two‐way ANOVA with Tukey's multiple comparisons test).

In stem cell therapy and tissue engineering, reducing oxidative stress with bioactive substances that can be released in a sustainable and controllable manner presents an effective strategy to mitigate stem cell aging and modulate inflammation.^[^ ^35^ ^]^ Such materials can enhance stem cell function and promote tissue regeneration by providing a consistent therapeutic environment and prolonging effects. The slow disassembly of the PVA‐GA hydrogel in physiological media, driven by solvation and disruption of hydrogen bonding, would allow for the sustained release of GA. To understand the release dynamics, we monitored the release of GA from hydrogels with varied compositions (GA 2 wt.%, 1 wt.%, and 0.5 wt.%) over a period of 14 d in distilled water. As expected, the concentration‐dependent release profile of GA was observed. The release of GA from the hydrogel slowed down with the decrease in GA concentration. The cumulative release of GA from hydrogels with different GA concentrations reached 14.53%, 13%, and 6.56% over 14 d, respectively (**Figure** **3**A).

These results indicate that the release profile of GA can be controlled by adjusting its concentration to meet specific biomedical requirements. The sustained release of GA from hydrogels may enable its long‐term activity in cell culture, maintaining effective GA concentrations while minimizing the undesired side effects associated with high GA concentrations. In comparison to directly using a GA solution, incorporating GA into the hydrogel would avoid the need for repeated GA applications, making it more suitable for use as an implant material.

### 2.4 Pro‐oxidative and Antioxidative Properties of GA

Given the dual pro‐oxidant and antioxidant properties of GA,^[^ ^19^, ^26^ ^]^ we first explored how these attributes are influenced by factors such as GA concentration, ROS levels, hydrogen bonding between PVA and GA, and encapsulation within a hydrogel matrix.

For pure GA, both autoxidation and antioxidant activities exhibited a positive correlation with increasing concentrations (Figure S4A,B, Supporting Information). This concentration‐dependent enhancement of GA's dual activities can be attributed to the reactivity of its phenolic hydroxyl groups. In autoxidation, GA's pro‐oxidant behavior originates from the oxidation of its hydroxyl groups by oxygen, forming quinone‐like structures and generating ROS. At higher GA concentrations, more molecules are available for reaction, leading to greater ROS production. Likewise, GA's antioxidant activity—involving the donation of electrons or hydrogen atoms from its phenolic hydroxyl groups to neutralize free radicals or reduce oxidizing agents—also increases with concentration.

To investigate how the behavior of GA varies under low and high ROS conditions, its antioxidation capacity was quantified under varying levels of oxidative stress. H_2_O_2_/Fe^2+^ mixtures were employed to generate highly reactive hydroxyl radicals (·OH), simulating elevated oxidative stress conditions. We found that when H_2_O_2_ and Fe^2+^ concentrations remained below a critical threshold (H_2_O_2_/GA molar ratio of 2.5), the total ROS levels stayed consistently low, comparable to the control group (GA solution without H_2_O_2_/Fe^2+^). However, once H_2_O_2_ and Fe^2+^ concentrations surpassed this threshold, ROS levels in the system increased sharply, indicating that GA's antioxidant capacity was overwhelmed by escalating oxidative stress (Figure S4C, Supporting Information). This observation can be attributed to the interplay between GA's antioxidant mechanisms and the ROS generation dynamics. At low H_2_O_2_/Fe^2+^ concentrations, ·OH production is limited and can be effectively neutralized by GA. As H_2_O_2_/Fe^2+^ concentrations surpass the threshold, the generation of ·OH exceeds GA's neutralization capacity, resulting in a rapid accumulation of total ROS. These findings indicate that GA exhibits robust antioxidant activity under low ROS levels, underscoring its potential in biomedical applications in mild oxidative environments.

We subsequently investigated whether PVA‐GA hydrogen bonding influences the pro‐oxidant and antioxidant properties of GA in solution. Compared to pure GA, the autoxidation level of PVA/GA mixtures decreased slightly only when the PVA‐to‐GA hydroxyl group molar ratio exceeded a threshold of 0.86 (Figure S5A, Supporting Information). These findings suggest that hydrogen bonding with PVA may stabilize GA, thereby reducing the reactivity of its hydroxyl groups with O_2_. This effect likely arises from a dense hydrogen‐bonding network at elevated PVA levels, which subtly restricts GA's molecular mobility or shields its reactive sites. In contrast, the antioxidation capacity of GA was not impacted by PVA across all tested ratios (Figure S5B, Supporting Information). The consistent antioxidant capacity of PVA/GA mixtures indicates that hydrogen bonding with PVA does not substantially hinder the electron‐donating ability of GA's phenolic hydroxyl groups. In summary, PVA‐GA hydrogen bonding subtly modulates GA's pro‐oxidant behavior at elevated PVA concentrations without compromising its antioxidant function. These interactions appear to be subtle and dynamic, preserving the availability of GA's functional hydroxyl groups for both pro‐oxidant and antioxidant roles.

To examine how a hydrogel matrix influences GA's pro‐oxidant and antioxidant properties, we prepared PVA‐GA hydrogels and compared them with pure GA solutions containing equivalent GA amounts. Relative to pure GA, PVA‐GA hydrogels exhibited reduced autoxidation levels (Figure S6A, Supporting Information). This observation aligns with findings from PVA/GA solutions, reflecting the high molar ratios of PVA hydroxyl groups to GA phenolic hydroxyl groups in the hydrogels (25.7, 12.9, and 6.4 for 0.5, 1, and 2 wt.% GA, respectively). The antioxidant capacity was assessed using an Antioxidant Assay Kit, which quantifies the reduction of Cu^2+^ to Cu^+^ by antioxidants. A significant reduction in detectable Cu^+^ was observed in the PVA‐GA hydrogel system compared to pure GA (Figure S6B, Supporting Information). Notably, the hydrogel with 0.5 wt.% GA, which has a higher PVA/GA ratio than those with 1 and 2 wt.% GA, exhibited a more pronounced decrease in Cu^+^ at early time points (3 and 5 min), suggesting that increased PVA content restricts Cu^2+^ access to GA. The polymer network in the hydrogel may adsorb Cu^2+^ ions, impede their diffusion to GA's phenolic hydroxyl groups, or partially immobilize GA, thereby reducing its availability for electron donation. Furthermore, lower Cu^+^ levels in the hydrogel groups at later time points (10–40 min) indicate that the hydrogel may also trap or adsorb Cu^+^, preventing its release into the solution for detection. Collectively, these results highlight the scavenging capabilities of PVA‐GA hydrogels, underscoring their potential for ROS sequestration.

### 2.5 Proliferation and Viability of hADSCs

The hydrogel, being a physically cross‐linked structure composed solely of PVA and GA, inherently lacks cell adhesion sites, as demonstrated by the poor cell adhesion and spreading (Figure S7A, Supporting Information). In this study, to investigate the effect of GA on hADSCs without interference from other components, the cells were co‐cultured with the pure hydrogel in an indirect‐contact manner, meaning they adhered to the surface of the tissue culture plate (TCP) rather than directly to the hydrogel. However, it is noteworthy that with a simple surface modification, the hydrogel can facilitate direct cell culture. For instance, coating the hydrogel surface with fibronectin, a typical extracellular matrix protein that facilitates cell attachment, enabled cell attachment and viability comparable to that on TCP (Figure S7, Supporting Information).

The effect of GA on cells is both concentration‐ and duration‐dependent, with higher dosages exhibiting cytotoxicity.^[^ ^21^ ^]^ Given the sustained release of GA from the gel (Figure 3A), we hypothesized that the hydrogel would maintain a lower concentration of GA in the culture medium compared to the direct addition of a GA solution. This would create a more moderate environment conducive to supporting cell survival. To verify this hypothesis, the cells were co‐cultured with hydrogels containing 1 wt.% GA for 7 d. The cell growth curves revealed that at a lower hydrogel dose (≤ 4 µL well^−1^ of a 24‐well TCP), the cells displayed a typical proliferation curve with no significant impact. However, cell proliferation was significantly inhibited when the hydrogel dose exceeded 8 µL well^−1^ (Figure 3B). Compared to the hydrogel, pure GA with an equal amount of GA showed a higher inhibitory effect on cell proliferation (Figure 3C). A significant inhibition of cell proliferation was observed when the GA concentration reached 40 µg mL^−1^, corresponding to an equivalent amount of 8 µL of hydrogel per well. These results support our hypothesis that the sustained release of GA from the hydrogel reduces cytotoxicity.

**Figure 4:**
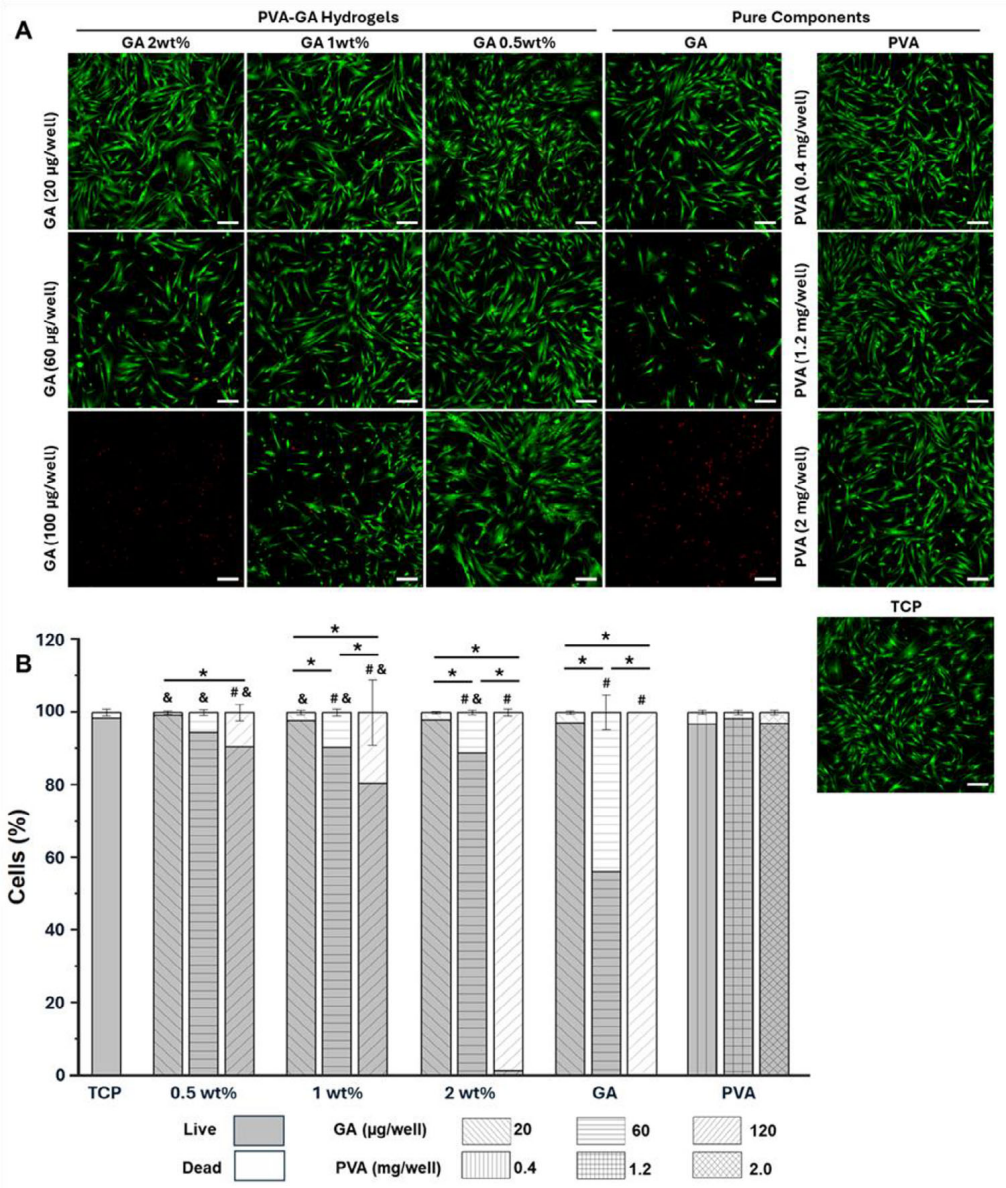
hADSC viability on hydrogels and in the presence of pure GA and PVA. A) Live (green)/Dead (red) staining of hADSCs after 48 h of culture with hydrogels of varying dosages and GA concentrations. Pure GA and PVA were included for comparison. The amount of GA (for the hydrogel and pure GA groups) and PVA (for the pure PVA group) in each well of standard 24‐well TCP is indicated; scale bar = 200 µm. B) Quantification of live and dead cells under different conditions. For each group, 5 images were analyzed. Statistical significance (*p* < 0.05, one‐way ANOVA with Tukey's multiple comparisons test) is indicated by * for comparisons of different GA or PVA dosages within each group, # for comparisons to the TCP control, and & for comparisons between the hydrogel and pure GA groups with the same GA amount.

Next, we examined the cell viability by co‐culturing hADSCs with the hydrogels, pure GA, and PVA for 48 h. According to the Live/Dead staining results (**Figure** **4**), cell viability decreased with the increase in GA dose. For both the hydrogel group containing 2 wt.% GA and the pure GA group, almost no living cells were detected at the GA dose of 100 µg well^−1^. However, the cytotoxicity of the phenolic molecule was diminished in the hydrogels with 1 wt.% and 0.5 wt.% GA, showing viable cells even at the high GA dose. This observation could be explained by the lower GA release rate in such hydrogels, which decreased the local GA concentration that the cells were exposed to. Such an effect of GA release on cell survival could be further confirmed by the groups with a GA dose of 60 µg well^−1^, as more viable cells were detected when the GA concentration in the hydrogel decreased. In line with the literature that PVA has a high cell compatibility,^[^ ^36^ ^]^ we did not observe cytotoxicity from PVA. These results indicate that the cytotoxicity of PVA‐GA hydrogel is primarily attributable to GA rather than PVA. It has been reported that gallic acid undergoes autoxidation, particularly at relatively high concentrations, leading to ROS‐dependent cell death.^[^ ^37^ ^]^

Utilizing a hydrogel with low GA concentrations would slow the release of GA, minimize the peak concentration of GA, and thereby reduce the associated cytotoxic effect. Therefore, optimizing the GA concentration within the hydrogel could be an easy way to achieve the desired release profile, enhancing the biological functions of hydrogels for long‐term cell culture and tissue engineering applications. Such controlled release mechanisms are especially beneficial for applications requiring sustained delivery of bioactive molecules to support stem cell survival and tissue regeneration.

### 2.6 PVA‐GA Hydrogel Removes ROS

ROS‐induced oxidative stress is one of the primary factors regulating cell senescence and increasing susceptibility to senescence‐related diseases.^[^ ^38^, ^39^ ^]^ As primary cells, MSCs are particularly sensitive to oxidative stress, presenting stagnated proliferation, elevated senescence, mitochondrial dysfunction, and altered differentiation potential when exposed to high levels of ROS.^[^ ^15^, ^39^ ^]^

It is known that GA, a natural polyphenol, can donate hydrogen atoms from its phenolic hydroxyl groups to free radicals, converting them into more stable and less reactive molecules.^[^ ^40^ ^]^ Therefore, we anticipated that the strong antioxidative activity of GA could endow the PVA‐GA hydrogel with a high capacity for ROS scavenging, thereby mitigating ROS‐induced MSC senescence and preserving their functionality.

**Figure 5:**
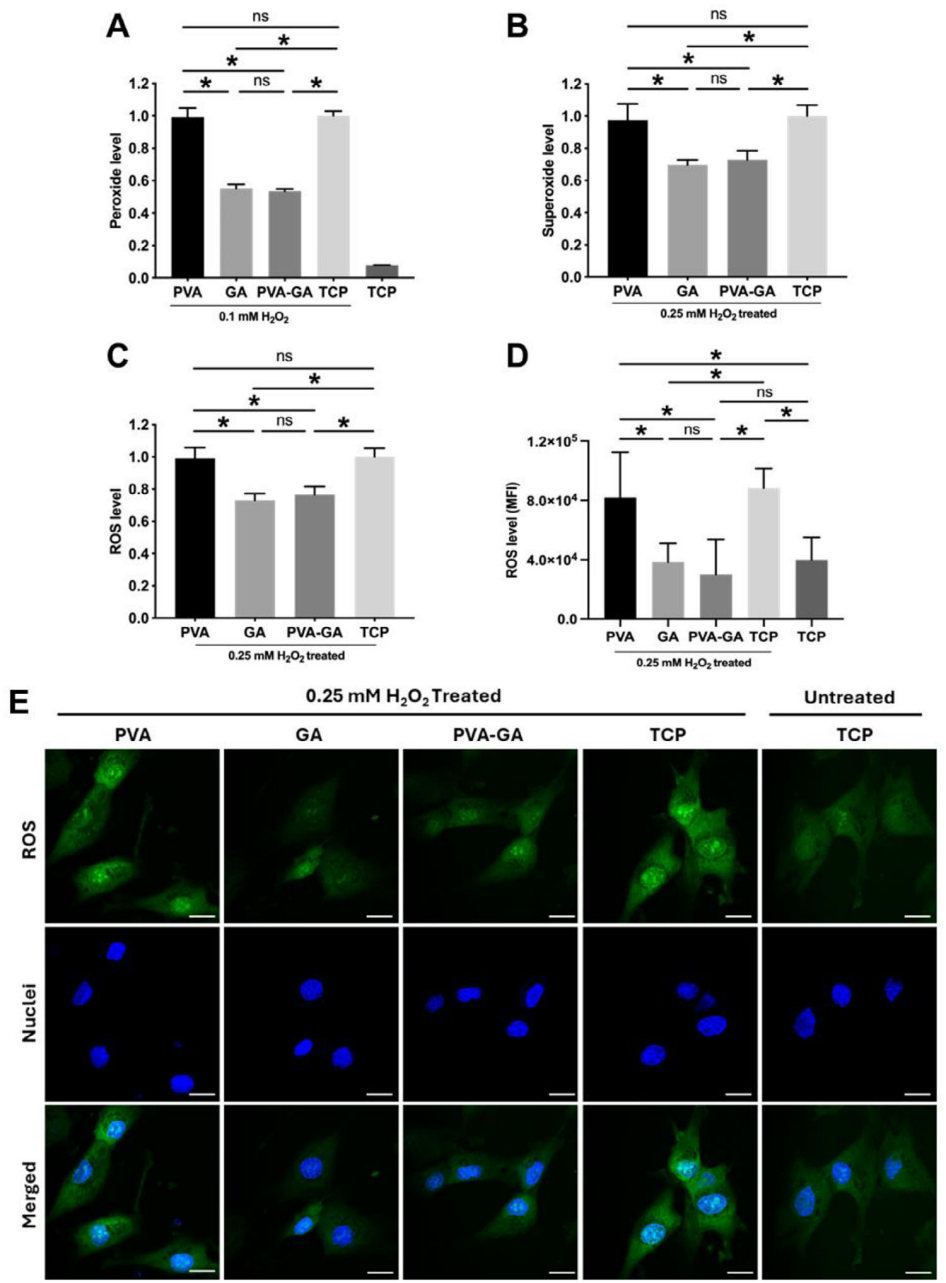
Antioxidant effect of PVA‐GA hydrogel. A) Removal of peroxide by the PVA‐GA hydrogel in a cell‐free environment. Samples were incubated at 37 °C with 0.1 mM H_2_O_2_ for 2 h, followed by quantification of peroxide remaining in the solution (n ≥ 3, *p < 0.05, one‐way ANOVA with Tukey's multiple comparisons test). B,C) Quantification of intracellular superoxide and ROS in hADSCs after 2 h of treatment with 0.25 mM H_2_O_2_, in the presence of PVA‐GA hydrogel, pure PVA, and GA (n ≥ 3, *p < 0.05, one‐way ANOVA with Tukey's multiple comparisons test). D,E) Quantification of mean fluorescence intensity (MFI) of cellular ROS D) based on live‐cell staining images E); scale bar = 20 µm (n ≥ 6, *p < 0.05, one‐way ANOVA with Tukey's multiple comparisons test).

We first assessed the ROS scavenging capacity in a cell‐free test by incubating the hydrogel (GA 1 wt.%), as well as an equivalent amount of pure GA and PVA, in 0.1 mM H_2_O_2_ for 2 h. Compared to the TCP positive control, both the PVA‐GA hydrogel and pure GA significantly reduced peroxide levels (**Figure** **5**A). The peroxide level in the PVA group was similar to that in the positive control, indicating that GA, not PVA, is responsible for removing ROS in the hydrogel. Notably, according to GA release kinetics, only less than 10% of GA was released within 3 d, meaning that most of the GA molecules in the PVA‐GA hydrogel remained in an “encapsulated” form during the test. These results indicate that both encapsulated and released GA can scavenge ROS effectively.

Further, we assessed whether the hydrogel could effectively remove ROS in a cell culture environment. To simulate a high oxidative stress microenvironment, H_2_O_2_ was added to the hADSC culture medium (FBS‐free) to achieve a final concentration of 0.25 mM. After 2 h of treatment, the medium was replaced, and the levels of superoxide and total ROS in the cells were measured after 24 h (Figure 5B,C). Additionally, live‐cell ROS staining was performed following the treatment described above to further evaluate ROS levels within the cells. Both the hydrogel and pure GA significantly reduced the intracellular superoxide and total ROS levels, whereas PVA did not exhibit ROS scavenging activity (Figure 5D,E).

**Figure 6:**
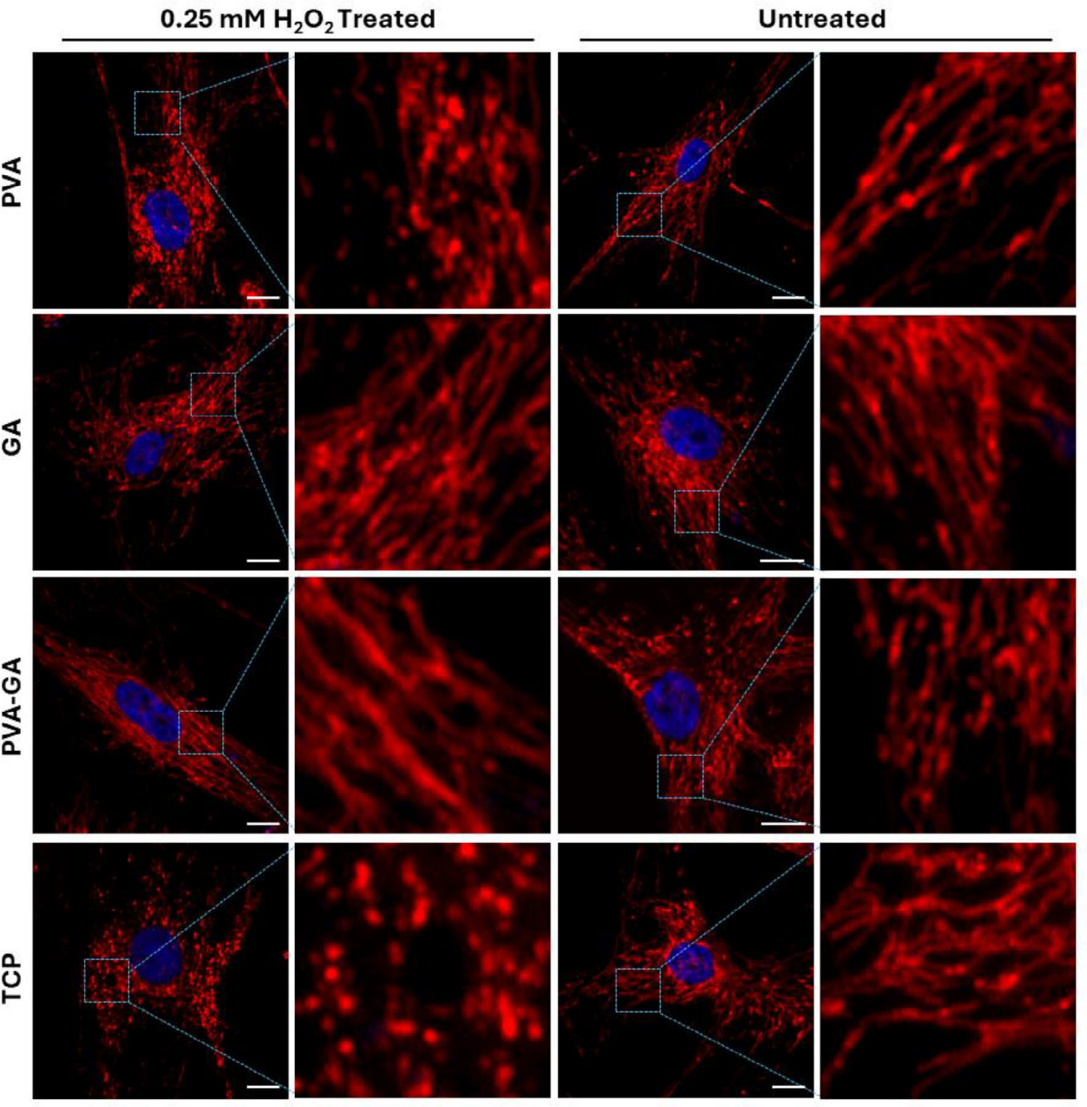
Mitochondrial integrity protection by PVA‐GA hydrogel. Representative live‐cell staining of mitochondria (red) and nuclei (blue) of hADSCs with or without H_2_O_2_ (0.25 mM) treatment, in the presence of PVA‐GA hydrogel, pure PVA, and GA; scale bar = 20 µm.

As reported, ≈80% of ROS are produced in mitochondria during aerobic cell metabolism, specifically through oxidative phosphorylation.^[^ ^41^ ^]^ Excessive ROS, whether generated by mitochondria or originating from the microenvironment, can cause mitochondrial damage. As the energy powerhouse of cells, mitochondrial dysfunction impairs the energy supply, affecting critical processes such as protein synthesis, repair, and overall cellular maintenance, which can accelerate cellular senescence and lead to cell death, resulting in chronic inflammation and contributing to various diseases.^[^ ^42^, ^43^, ^44^, ^45^ ^]^ Given the observed effective ROS scavenging capacity, we investigated whether the hydrogel could prevent mitochondrial damage caused by excessive ROS. After 2 h of H_2_O_2_ treatment, cells in the TCP and PVA groups exhibited significantly transformed and fragmented mitochondria, in contrast to the intact mitochondria in untreated cells. Conversely, H_2_O_2_ treatment did not alter the mitochondrial morphology in the PVA‐GA hydrogel and GA groups, which maintained their normal elongated shape (**Figure** **6**).

Since mitochondria are highly susceptible to oxidative damage,^[^ ^43^ ^]^ our results demonstrate that the PVA‐GA hydrogel could significantly protect hADSCs against oxidative stress. By preserving mitochondrial integrity and function, the PVA‐GA hydrogel could potentially enhance the longevity and functionality of hADSCs.

### 2.7 PVA‐GA Hydrogel Mitigates Cellular Senescence

After a limited number of divisions, primary cells enter a state of replicative senescence. It has been reported that MSCs exhibit an increased percentage of senescent cells during in vitro culture, with associated abnormal morphology and proliferation arrest after multiple passages.^[^ ^46^, ^47^ ^]^ Oxidative stress is considered a primary cause of cellular senescence, as it induces DNA damage, mitochondrial dysfunction, and activation of signaling pathways that regulate growth arrest.^[^ ^48^, ^49^ ^]^

**Figure 7:**
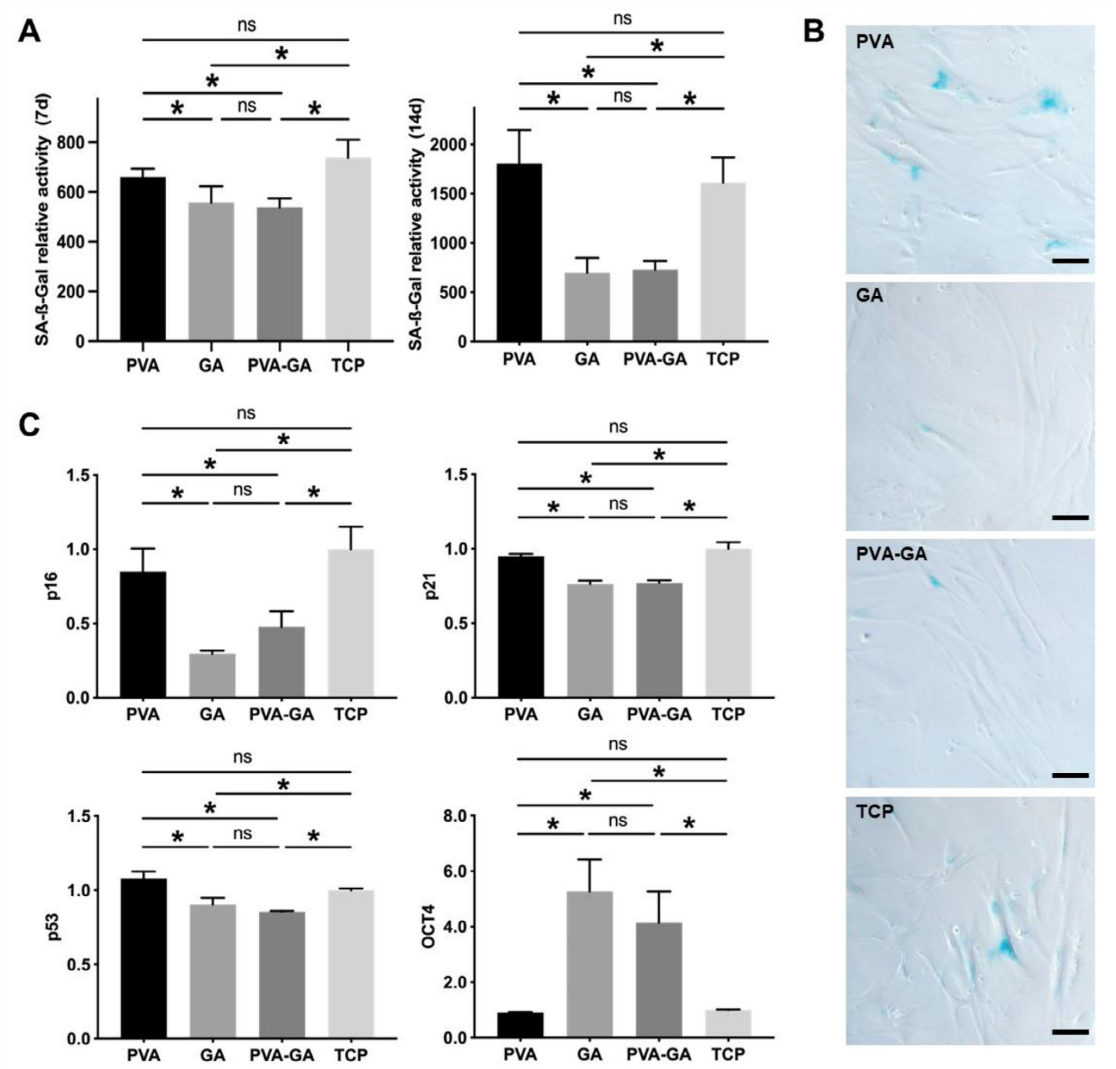
Anti‐senescence effect of PVA‐GA hydrogel. A) Relative SA‐β‐Gal activity in hADSCs after 7 and 14 d of culture, in the presence of PVA‐GA hydrogel, pure PVA, and GA (n ≥ 3, *p < 0.05, one‐way ANOVA with Tukey's multiple comparisons test). B) Representative SA‐β‐Gal staining (blue) images of hADSCs after 14 d of cultivation under different conditions; scale bar = 200 µm. C) Real‐time PCR quantification of p53, p21, p16, and OCT4 mRNA in hADSCs cultured under different conditions. The values of cells cultured on TCP were normalized to 1 (n ≥ 3, *p < 0.05).

Based on the observed ROS scavenging capacity and mitochondrial protection effect of the PVA‐GA hydrogel, we next assessed the cellular senescence in hADSCs by quantifying the activity of SA‐β‐Gal, a widely used biomarker for identifying and measuring cellular senescence. The senescent hADSCs at passage 11 were cultured with the PVA‐GA hydrogel, as well as pure GA and PVA in an equivalent amount to that in the hydrogel. As shown in both the quantification (**Figure** **7**A) and the staining (Figure 7B) of SA‐β‐Gal, the PVA‐GA hydrogel and pure GA significantly mitigated hADSC senescence after 7 d and 14 d of culture, which could be attributed to their ROS scavenging effect. In contrast, cells cultured with pure PVA exhibited similar levels of SA‐β‐Gal to those of control cells in TCP, consistent with the observation that PVA alone was ineffective in removing ROS.

As has been reported, mitochondrial ROS can induce DNA damage and accelerate telomere‐induced senescence.^[^ ^50^, ^51^ ^]^ Therefore, reducing mitochondrial ROS is known to extend the replicative lifespan of cells.^[^ ^52^ ^]^ In this senescence assay, no additional oxidative stress (H_2_O_2_ treatment) was applied to the cells. Consequently, the intracellular ROS were primarily generated by mitochondria. These results, combined with the demonstrated antioxidant and mitochondrial protective effects of the PVA‐GA hydrogel, suggest that the anti‐senescence mechanism of the PVA‐GA hydrogel likely involves ROS scavenging, prevention of mitochondrial oxidative damage, and maintenance of mitochondrial integrity and function.

Cellular senescence can arise in response to various forms of cellular stress or damage, which activate the DNA‐damage response (DDR) pathways. This activation, in turn, triggers the p53/p21 and p16 signaling cascades, leading to cell cycle arrest and the establishment of a senescent state.^[^ ^53^ ^]^ In this study, we evaluated the mRNA levels of p53, p21, and p16 in the cells and observed that the presence of the hydrogel significantly downregulated the expression of all three senescence genes. Moreover, cells in the hydrogel and GA groups maintained higher expression levels of OCT4, a critical stemness gene essential for maintaining pluripotency and promoting self‐renewal in stem cells (Figure 7C). These findings highlight the potential role of the hydrogel in mitigating cellular senescence at the genetic level.

### 2.8 Hydrogel Mitigated the Effects of ROS on Cell Differentiation

**Figure 8:**
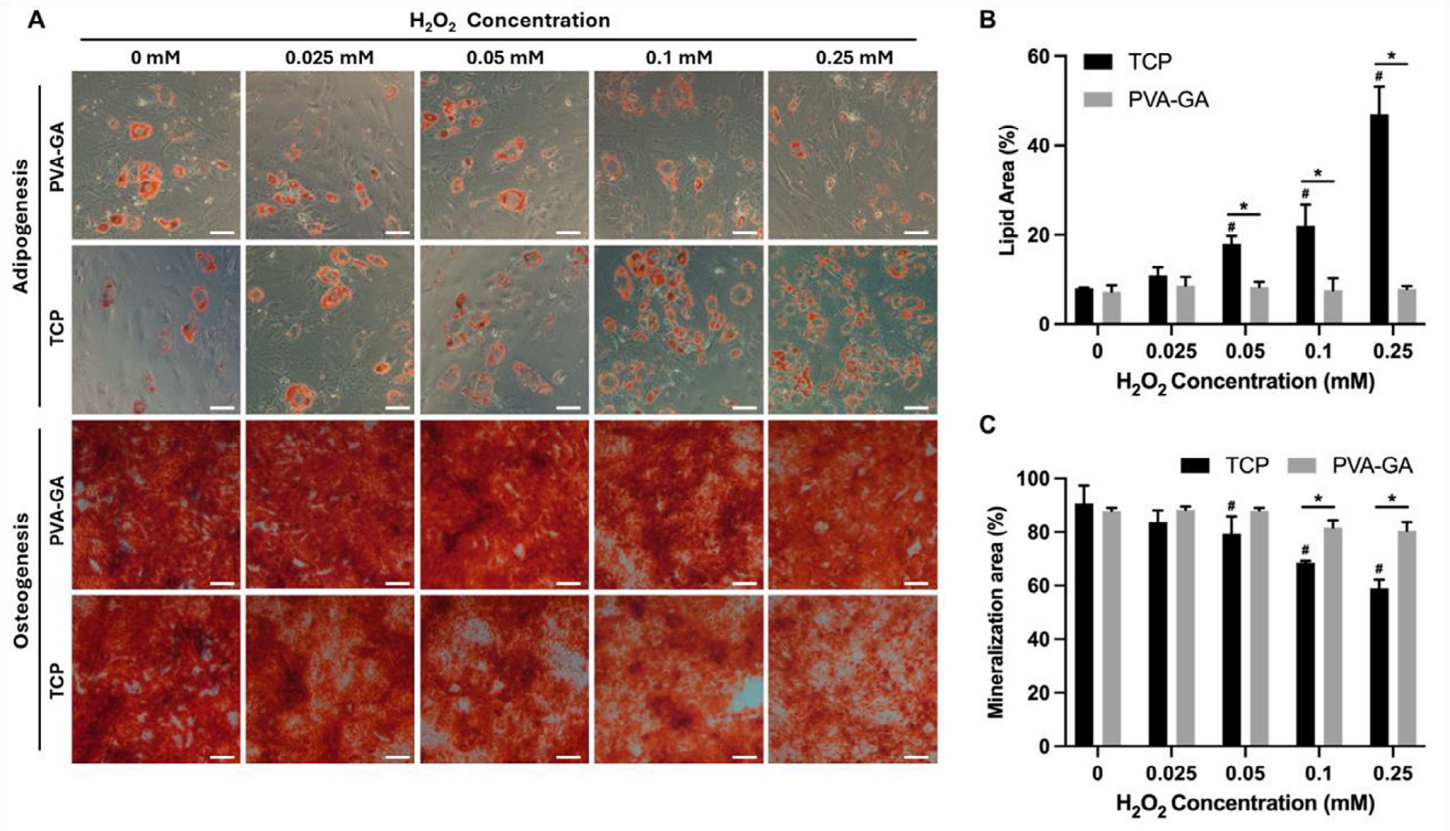
Differentiation capacity of hADSCs under varying concentrations of H_2_O_2_. A) Oil Red O staining (red) for adipogenesis (scale bar = 50 µm) and Alizarin Red S staining (red) for osteogenesis (scale bar = 200 µm) of hADSCs cultured with and without PVA‐GA hydrogel. B) Quantitative analysis of adipogenic differentiation (lipid area) and osteogenic differentiation (mineralization area) under varying H_2_O_2_ treatments, comparing the TCP and hydrogel groups (n ≥ 3, *p < 0.05, two‐way ANOVA with Tukey's multiple comparisons test). * Indicates a significant difference between the TCP and hydrogel groups (*p < 0.05), while # indicates a difference compared to the non‐H_2_O_2_‐ treated TCP group (^#^ *p* < 0.05).

Stem cell differentiation potential is one of the most important characteristics, playing a crucial role in stem cell therapy. It is important to note that elevated levels of ROS can not only lead to cellular damage and dysfunction but also influence cell differentiation. Studies have reported that higher levels of ROS can stimulate adipogenic differentiation of MSCs while inhibiting osteogenesis.^[^ ^13^, ^15^ ^]^ In this study, we treated cells with different concentrations of H_2_O_2_ (ranging from 0 to 0.25 mM) during the differentiation induction process, to observe the effects of ROS on stem cell differentiation with and without hydrogel. In line with the literature, increasing concentrations of H_2_O_2_ promoted adipogenic differentiation while inhibiting osteogenic differentiation in the TCP control group. Conversely, in the presence of the hydrogel, both adipogenic promotion and osteogenic inhibition were reduced, indicating that the hydrogel mitigated the effects of elevated ROS levels on differentiation (**Figure** **8**). Additionally, flow cytometry analysis revealed that typical phenotypic markers of MSCs, including CD271, CD73, CD90, and CD105, were well maintained in the cells cultured with the hydrogel for 14 d compared to control cells (Figure S8 and Table S1, Supporting Information).

Taken together, the results indicate that the PVA‐GA hydrogel effectively mitigated the effects of elevated ROS levels on differentiation, while also preserving the phenotype and multi‐lineage differentiation potential of hADSCs, highlighting its good stem cell compatibility for extended culture periods.

### 2.9 PVA‐GA Hydrogel Preserves Pro‐angiogenic Effect of hADSCs under Oxidative Stress

**Figure 9:**
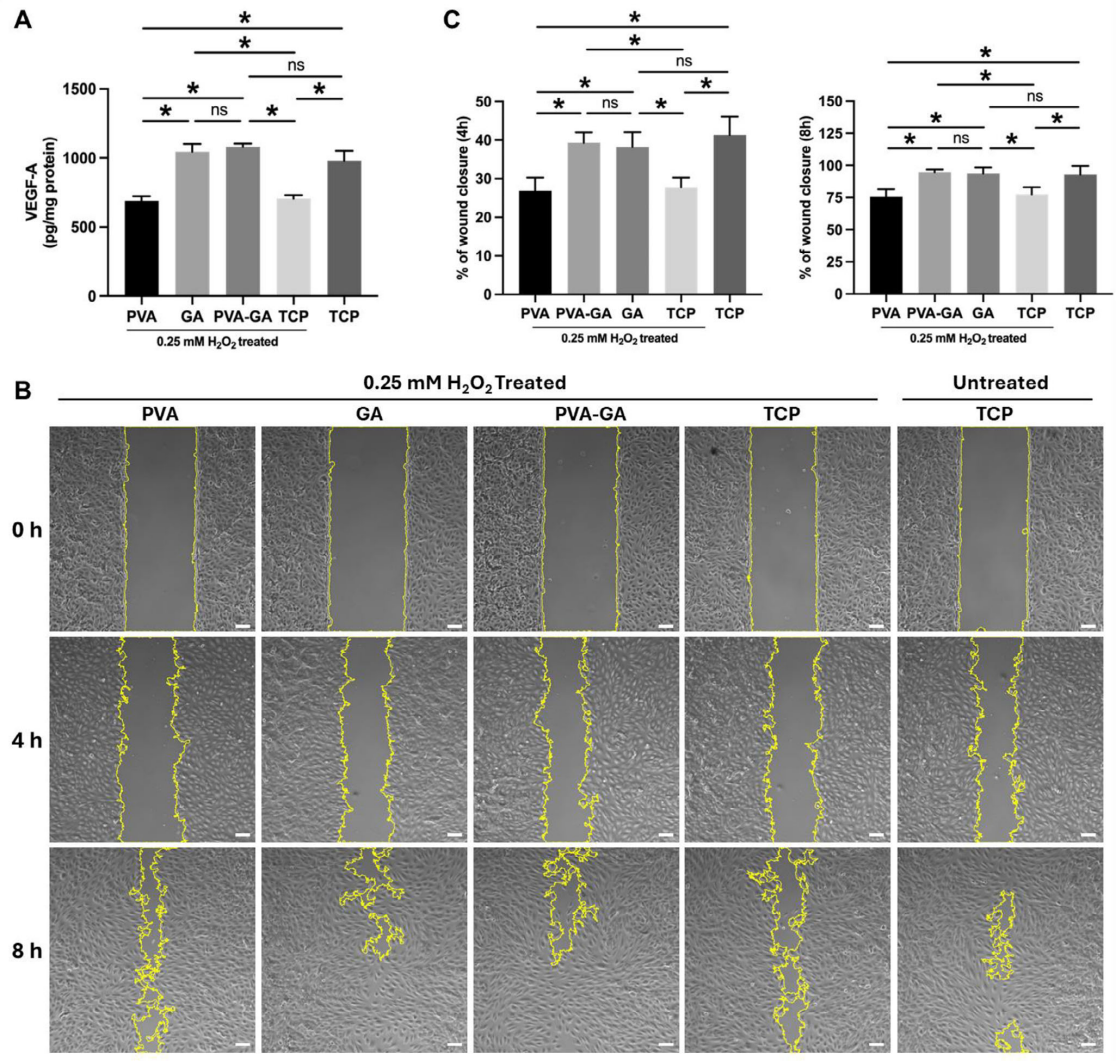
Effect of PVA‐GA hydrogel on VEGF secretion from hADSCs and its impact on HUVECs migration. A) Quantification of VEGF‐A in the conditioned medium derived from hADSCs cultured under various conditions (n ≥ 3, *p < 0.05, two‐way ANOVA with Tukey's multiple comparisons test). B) Representative images of HUVECs at 0, 4, and 8 h after gap creation, cultured with various conditioned medium; scale bar = 100 µm. C) The percentage of wound closure was measured to assess cell migration, with quantitative analysis presented for each time point (*n* = 8, *p < 0.05, two‐way ANOVA with Tukey's multiple comparisons test).

The paracrine effects of stem cells are among the most critical mechanisms by which stem cell therapy achieves its therapeutic outcomes.^[^ ^54^, ^55^ ^]^ Among the secretome of MSCs, VEGF plays a central role in key processes such as angiogenesis, tissue repair, and wound healing.^[^ ^56^ ^]^ Exposure of stem cells to low doses of oxidative stress can enhance VEGF paracrine secretion, promoting angiogenesis and tissue repair. Conversely, higher levels of oxidative stress markedly suppress VEGF secretion, impairing the regenerative potential of stem cells and disrupting their therapeutic efficacy.^[^ ^57^, ^58^ ^]^ To investigate the potential protective effects of hydrogels on VEGF secretion from cells under oxidative stress, we treated hADSCs with H_2_O_2_ (0.25 mM) for 2 h. After 7 days of culture, the supernatants were collected to assess VEGF secretion levels, which were then used as conditioned medium for subsequent experiments. The results indicated that H_2_O_2_ treatment significantly reduced VEGF secretion in cells cultured on TCP. However, in the presence of the hydrogel and GA, the cells maintained higher VEGF secretion levels, comparable to those of untreated cells. These results suggest that the hydrogel and GA preserve VEGF secretion under oxidative stress, potentially by scavenging ROS from the environment (**Figure** **9**A).

**Figure 10:**
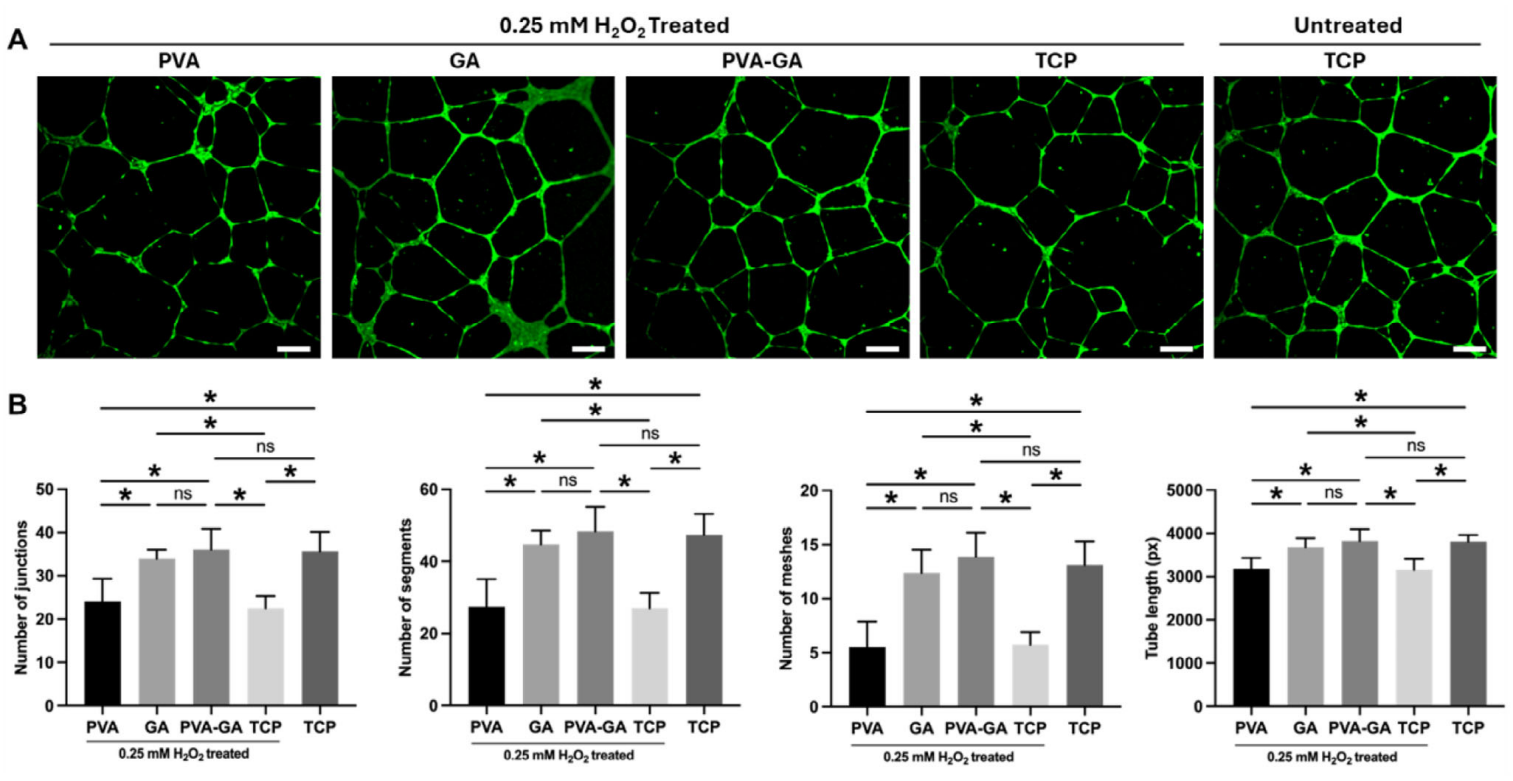
The impact of VEGF secretion from hADSCs on HUVECs angiogenesis. A) Representative images of HUVECs on Geltrex, cultured with various conditioned medium. Capillary‐like structures were observed; scale bar = 100 µm. B) Quantitative analysis was performed to measure total tube length, number of junctions, segments, and meshes (*n* = 8, *p < 0.05, two‐way ANOVA with Tukey's multiple comparisons test).

Next, we utilized the conditioned medium derived from hADSCs cultured under different conditions to culture HUVECs. The results of the wound healing assay demonstrated that HUVECs, in the conditioned medium from the hydrogel and GA groups, exhibited significantly faster migration compared to cells in conditioned medium from the PVA and TCP groups (Figure 9B,C). Similarly, the tube formation assay revealed that the conditioned medium from the hydrogel and GA groups with H_2_O_2_ treatment effectively stimulated tube formation in HUVECs (**Figure** **10**). Together, these findings demonstrate the ability of the PVA‐GA hydrogel to preserve the pro‐angiogenic capacity of MSCs under oxidative stress.

## 3 Conclusion

In this study, PVA‐GA supramolecular hydrogels were synthesized in a simple, eco‐friendly, and cost‐effective manner. These hydrogels address the challenge of protecting stem cells from oxidative stress and senescence. A key innovation of our approach is the sustained release of GA from the hydrogel, enabling extended biological activity. The PVA‐GA hydrogel not only mitigates cellular senescence in MSCs by alleviating oxidative stress, but also maintains mitochondrial integrity, preserves VEGF secretion and the multipotency of differentiation under elevated oxidative stress.

Our findings highlight the potential of utilizing natural polyphenol‐based hydrogels for stem cell protection and therapy. Although the physically cross‐linked hydrogel lacks inherent cell adhesion sites, modifications such as adding coatings or grafting cell‐adhesive molecules can facilitate direct contact culture with cells. These modifications can enhance cell adhesion and pave the way for advanced biomedical applications of such hydrogels in diverse areas such as wound healing, tissue engineering, and 3D bioprinting. Additionally, the diverse beneficial properties of GA, such as its antibacterial, anticancer, anti‐inflammatory, and immunomodulatory effects, present promising opportunities for further research. By optimizing the component ratios, the mechanical properties of these hydrogels can be tailored to meet specific application needs. Our study not only demonstrates the effectiveness of PVA‐GA hydrogels in enhancing MSCs biological functions but also offers a promising strategy for advancing stem cell therapies and exploring their broader therapeutic potential.

## 4 Experimental Section

### PVA‐GA Hydrogel Preparation and Characterization

PVA (Mw = 145 kDa, 99.0–99.8 mol%, Merck, Darmstadt, Germany) and GA (≥ 99.0%, Merck, Darmstadt, Germany) were first dissolved in water at 90 °C under magnetic stirring. The resulting homogeneous solutions were then cooled to 4 °C until spontaneous gelation occurred. To prepare hydrogels with varied PVA:GA ratios, the PVA was fixed at 10 wt.% while GA concentrations were varied (5 wt.%, 2 wt.%, 1 wt.%, and 0.5 wt.%).

The FTIR spectra of the hydrogels and pure compounds were recorded in their dry state using a Nicolet iN10 infrared microscope (Thermo Fisher Scientific, Schwerte, Germany). The spectral range covered was 500–4000 cm^−1^, with data collected over 30 scans at a resolution of 4 cm^−1^. The internal microstructure of the hydrogels was examined using SEM (Supra 40 VP, Carl Zeiss, Germany). The hydrogels were freeze‐dried and then immersed in liquid nitrogen for 2 min before cross‐sectioning. The samples were coated with gold and observed using a field emission scanning electron microscope (Carl Zeiss‐Sigma, Germany) at an acceleration voltage of 3 kV.

The textural properties of the samples were characterized by N_2_ adsorption‐desorption, performed at −196 °C in a Micromeritics ASAP 2020. Before the measurements, the samples were degassed at 30 °C for 48 h under vacuum. From N_2_ adsorption‐desorption isotherms, the total pore volumes were calculated at a relative pressure P/P_0_ = 0.99.

A rheological analysis was performed to assess the viscoelastic properties of the hydrogels using a Physica MCR 301 rotational rheometer (Anton Paar, Graz, Austria) equipped with an 8 mm parallel plate geometry. The samples were prepared as 8 mm diameter discs. Amplitude sweeps were conducted from 0.01% to 40% strain at a constant angular frequency of 10 rad s^−1^ and a temperature of 20 °C to identify the LVR. Following this, frequency sweeps were performed at a constant strain of 0.5%, over a range of 0.1 to 100 rad s^−1^, at 20 °C. Additionally, temperature sweeps were performed from 5 to 60 °C, with a heating rate of 2 °C min^−1^, at a fixed frequency of 10 rad s^−1^ and 0.5% strain to determine the gel‐sol transition. All samples were tested in duplicate, and representative curves were presented. The mechanical properties of the hydrogels were evaluated at 25 °C using an Instron Universal Testing Instrument (model 3342) with a 50 N load cell. Rectangular specimens (15 mm wide, 60 mm long, and 1 mm thick) were used for uniaxial elongation tests with a constant stretching speed of 60 mm min^−1^. Stress relaxation measurements involved applying a fixed strain of 50% for 600 s while recording the time‐dependent stress. Each sample was tested with six specimens, and the average values were recorded.

The adhesive properties of the hydrogels were assessed using the probe tack method on an Instron Universal Testing Instrument (model 3342). Samples with a diameter of 1 cm and a thickness of 3 mm were tested. This method involved applying a 6 nN compression force for 5 s using a stainless‐steel flat‐ended probe with a diameter of 10 mm. The force required to detach the probe from the sample was quantified. The maximum force observed during detachment indicated the tackiness of the hydrogel, while the area under the stress‐strain curve during this phase represented the adhesion energy. The nanomechanical property of the hydrogel surface was further characterized using an MFP‐3D AFM (Asylum Research, Oxford Instruments, UK) at room temperature in contact mode. A silicon cantilever (OLYMPUS OMCL TR400 PB, Japan) with a pyramidal tip shape, a tip radius of 7 nm, a half‐angle of 36 degrees, and a spring constant of 0.8 nN nm^−1^ was used. Measurements were performed with an indentation force (F) ranging from 1 to 10 nN. The average Young's modulus (E) was obtained based on 100 single indentations for each sample and calculated from the force‐distance curves according to the Hertz model using the software MountainsSPIP (Digital Surf, France).

To examine the release profile of GA from the hydrogel, 1 g of hydrogel was incubated in 2 mL of distilled water in a cell culture incubator. At the specified time points, 1 mL of supernatant was replaced with an equal volume of fresh distilled water, and the absorbance at 300 nm of the aspirated supernatant was measured immediately using a microplate reader (Infinite 200 Pro, Tecan Group Ltd., Männedorf, Switzerland). The GA concentration was then determined based on a standard curve, established by measuring the absorbance of GA solutions with known concentrations.

### Pro‐oxidative and Antioxidative Properties of GA

To study GA autoxidation, aqueous GA solutions (20–160 µg mL^−1^) were incubated at 37 °C for 24 h. H_2_O_2_ levels were measured using the Amplex Red Hydrogen Peroxide/Peroxidase Assay Kit (Thermo Fisher Scientific, Schwerte, Germany) according to the manufacturer's instructions. Total ROS levels were quantified with the OxiSelect In Vitro ROS/RNS Assay Kit (Cell Biolabs, San Diego, USA) following the provided protocol. To explore the effect of hydrogen bonding between PVA and GA on autoxidation of GA, solutions with varying PVA/GA ratios were prepared, maintaining GA at 80 µg mL^−1^ and PVA at 4–1600 µg mL^−1^. Controls included pure water, PVA (62 µg mL^−1^), and GA (80 µg mL^−1^). Additionally, PVA‐GA hydrogels with different GA concentrations were tested for autoxidation. Hydrogels containing 80 µg GA were incubated in 1 mL water at 37 °C for 24 h, and total ROS levels in the solution were measured using the OxiSelect kit. Controls were pure water, PVA (800 µg mL^−1^), and GA (80 µg mL^−1^).

The antioxidant capacity of GA was evaluated at concentrations of 20–160 µg mL^−1^ using the Antioxidant Assay Kit (Thermo Fisher Scientific, Schwerte, Germany), which quantifies total antioxidant capacity by measuring the reduction of Cu^2+^ to Cu^+^ by antioxidants. To assess performance under varying ROS conditions, solutions of GA (200 µg mL^−1^), H_2_O_2_ (variable concentrations), and FeCl_2_ (H_2_O_2_: Fe^2+^ molar ratio of 30:1) were prepared. The amount of GA was kept constant across all solutions to ensure that any observed changes in ROS levels were due to varying oxidative stress rather than GA concentration. The amounts of H_2_O_2_/Fe^2+^ were varied to generate oxidative stress from low to high. Fe^2+^ was introduced to trigger the Fenton reaction, generating highly reactive hydroxyl radicals. Total ROS levels were measured using a DCFH working solution. Briefly, DCFH‐DA (Abcam, Berlin, Germany) was hydrolyzed with 0.01 M NaOH in the dark at room temperature for 30 min, neutralized with phosphate buffer, and added to the mixture. After 10 min of incubation in the dark, fluorescence was recorded at 485/535 nm (excitation/emission) using a microplate reader (Infinite 200 Pro, Tecan Group Ltd., Männedorf, Switzerland). The influence of PVA‐GA hydrogen bonding on antioxidant capacity was evaluated using solutions maintaining GA at 80 µg mL^−1^ and PVA at 4–1600 µg mL^−1^, with controls of pure water, PVA (62 µg mL^−1^), and GA (80 µg mL^−1^). For PVA‐GA hydrogels, antioxidant capacity was measured in hydrogels with 0.5, 1, and 2 wt.% GA, prepared in 96‐well plates (4.8 µL well^−1^), using the Antioxidant Assay Kit. Pure GA solutions with equivalent GA amounts served as controls.

### hADSC Culture

The hADSCs were isolated from lipoaspirate obtained following liposuction of subcutaneous adipose tissue. The collection of lipoaspirate and subsequent cell isolation were approved by the Ethics Committee of the University Hospital of Regensburg, Germany, under protocol number 08/117, following informed consent from the patient. The cells were maintained in a standard cell culture environment at 37 °C with 5% CO_2_, using Dulbecco's Modified Eagle Medium (DMEM; Thermo Fisher Scientific, Schwerte, Germany) supplemented with 10% fetal bovine serum (FBS; Sigma–Aldrich, Hamburg, Germany), 100 U mL^−1^ penicillin, and 100 µg mL^−1^ streptomycin (both from Merck Millipore, Darmstadt, Germany). The culture medium was refreshed every 3 d, and cells were passaged at a ratio of 1:3 upon reaching ≈90% confluence.

The effect of hydrogel and its pure components (PVA and GA) on hADSCs was examined, to understand their roles in regulating hADSCs. For the cells co‐cultured with hydrogel and pure PVA, a solution of PVA/GA or pure PVA was first dropped onto the bottom of TCP (TPP Techno Plastic Products AG, Trasadingen, Switzerland). After gelation at 4 °C for 30 min, cells were seeded onto the TCP surface. For co‐culture with GA, cells were first seeded onto the TCP, then the GA solution was added into the culture wells. For cells cultured directly on hydrogel, the TCP was first incubated with a PVA‐GA aqueous solution. After the water evaporated, a thin PVA‐GA hydrogel layer was formed for cell seeding and growth. For hydrogel with fibronectin coating, the hydrogel layer was incubated at 4 °C overnight with 10 µg mL^−1^ of fibronectin. 2 mL of culture medium was used for all groups, and the medium was refreshed every 3 d.

### HUVECs Culture

The cells were maintained in a standard cell culture environment at 37 °C with 5% CO_2_, using EGM‐2 Endothelial Cell Growth Medium (EGM; Lonza, Walkersville, USA). The culture plates were pre‐coated with the EmbryoMax 0.1% Gelatin Solution (Merck Millipore, Darmstadt, Germany). The culture medium was refreshed every 3 d, and cells were passaged at a ratio of 1:4 upon reaching ≈90% confluence.

### Cell Proliferation and Viability

The effect of hydrogel and pure GA on hADSC proliferation was assessed using Cell Counting Kit‐8 (CCK‐8; Dojindo, Japan). The cells (passage 6) were seeded at a density of 5.0 × 10^3^ cells/well into 24‐well TCP containing different doses of hydrogels or pure GA. From day 1 to day 7 post‐seeding, the medium was replaced with 350 µL of medium containing 10% CCK‐8 solution. After incubation at 37 °C for 2 h, the absorbance of the medium/CCK‐8 mixture at 450 nm was measured using a microplate reader (Infinite 200 Pro, Tecan Group Ltd., Männedorf, Switzerland). The cell number was calculated using a standard curve, which was established from various cell densities and corresponding absorbance values.

A Live/Dead assay was performed to assess the cytotoxicity of different components. Briefly, hADSCs (passage 6) were seeded into 24‐well TCP at a density of 1.0 × 10^4^ cells/well. The wells contained either hydrogels with different doses (containing 20, 60, 100 µg GA/well) or no hydrogel. For the PVA group, the wells contained PVA gel with varied PVA amounts (0.4, 1.2, 2 mg/well). For the GA group, after cell seeding, a GA solution (1 wt.%) was added to match the amount of GA present in the corresponding hydrogel. After 48 h of culture, the cells were stained with a Live/Dead Cell Imaging Kit (Thermo Fisher Scientific, Schwerte, Germany) following the provided protocol. Images were obtained using a confocal laser scanning microscope (LSM780, Carl Zeiss, Jena, Germany). Image analysis was performed using ImageJ software (version 1.54f, Wayne Rasband, National Institutes of Health, USA).

### ROS Removal Capacity of PVA‐GA Hydrogel

The capacity of the hydrogels to remove ROS was first examined in a cell‐free system. Hydrogels were prepared in a 24‐well TCP at a dose of 20 µL well^−1^ (GA 1 wt.%) and incubated with 0.1 mM H_2_O_2_ in distilled water at 37 °C. For comparison, groups containing pure PVA and GA, in amounts equivalent to those in the hydrogel, were included. TCP wells containing 0.1 mM H_2_O_2_ served as positive controls, while wells without H_2_O_2_ served as negative controls. After 2 h of incubation, the H_2_O_2_ concentration was assessed using the Amplex Red Hydrogen Peroxide/Peroxidase Assay Kit (Thermo Fisher Scientific, Schwerte, Germany).

To further evaluate the ability of the hydrogel to protect cells from oxidative stress by reducing excessive intracellular ROS levels in living cell cultures, hADSCs (passage 5) were seeded onto 24‐well TCP at a density of 1.0 × 10^4^ cells well^−1^. Consistent with the cell‐free test, the hydrogel (GA 1 wt.%) dose was 4 µL well^−1^, and pure PVA and GA were added in amounts equivalent to those in the hydrogel. The cells were treated with 0.25 mM H_2_O_2_ (prepared with FBS‐free DMEM) for 2 h, after which the medium was replaced. To assess ROS levels, the superoxide and total ROS in the cells were measured 24 h post‐treatment using a cell‐based ROS/Superoxide Detection Assay Kit (Abcam, Berlin, Germany) following the provided protocol. The fluorescence intensity of ROS and superoxide was quantified using a microplate reader (Infinite 200 Pro, Tecan Group Ltd., Männedorf, Switzerland). Additionally, to visualize ROS and nuclei in live cells, hADSCs were stained 24 h after H_2_O_2_ treatment using the CellROX Green Reagent Kit and Hoechst 33 342 (both from Thermo Fisher Scientific, Schwerte, Germany). Images were captured using a confocal laser scanning microscope (LSM780, Carl Zeiss, Jena, Germany) under consistent imaging settings across all samples. The MFI was quantified using ImageJ software (version 1.54f, Wayne Rasband, National Institutes of Health, USA). The total corrected fluorescence intensity within the region of interest (ROI) was then normalized by dividing it by the number of cells in the ROI.

### Cell Senescence

The senescence of hADSCs was examined by culturing the cells in the presence of hydrogel, pure PVA, and pure GA. For this assessment, hADSCs (passage 11) were seeded into 24‐well TCP at a density of 5.0 × 10^3^ cells well^−1^. The wells contained either 4 µL of hydrogel (GA 1 wt.%) or no hydrogel. In the PVA group, PVA solution (10 wt.%, 4 µL) was added to the culture plate and allowed to gel before cell seeding. In the GA group, after cell seeding, a GA solution (1 wt.%, 4 µl) was added to the wells. The amount of pure PVA or GA used was equivalent to that in the corresponding hydrogel. After 7 or 14 d of cultivation, the SA‐β‐Gal activity was quantified using a cellular senescence activity assay kit (Cell Biolabs, San Diego, USA). The fluorescence intensity of SA‐β‐Gal was normalized to the total protein content in the cell culture, measured with a BCA protein assay kit (Thermo Fisher Scientific, Schwerte, Germany). Additionally, senescent cells were identified by staining for SA‐β‐Gal at 37 °C overnight using a staining kit (Cell Signaling Technology, Frankfurt am Main, Germany). Images were captured using an Axiovert 40C microscope (Carl Zeiss, Jena, Germany).

To further investigate cell senescence, hADSCs (passage 11) were cultured for 14 days, and then the total RNA was isolated using a PureLink RNA kit (Thermo Fisher Scientific, Schwerte, Germany) following the given protocol. The cDNA was synthesized using the RevertAid First Strand cDNA Synthesis Kit (Thermo Fisher Scientific, Schwerte, Germany) and amplified using the PowerUp SYBR Green Master Mix (Thermo Fisher Scientific, Schwerte, Germany) on a real‐time PCR system (StepOnePlus, Thermo Fisher Scientific, Schwerte, Germany).

The expression of the following genes was quantified using real‐time PCR with the corresponding primers: OCT4 (5′‐ACATCAAAGCTCTGCAGAAAGAACT‐3′ and 5′‐CTGAATACCTTCCCAAATAGAACCC‐3′), p53 (5′‐TGACTGTACCACCATCCACTA‐3′ and 5′‐AAACACGCACCTCAAAGC‐3′), p21 (5′‐GAGACTCTCAGGGTCGAAAA‐3′ and 5′‐TTAGGGCTTCCTCTTGGAGA‐3′), and p16^INK4A^ (5′‐AGCATGGAGCCTTCGGCTGA‐3′ and 5′‐CCATCATCATGACCTGGATCG‐3′). GAPDH was used as a housekeeping gene to normalize the Δ*C*_t_ values of target genes (Δ*C*_t_  =  *C*_t_, target – *C*_t_, GAPDH). The fold change of expression (sample/control) was expressed as 2^–ΔΔ^ *^C^*_t_ (ΔΔ*C*_t_  = Δ*C*_t_, sample – Δ*C*_t_, control).

### hADSC Differentiation and Phenotype

hADSCs (passage 7) were seeded into the 24‐well TCP containing 4 µL of hydrogel (GA 1 wt.%) and cultured in growth medium. The TCP without hydrogel served as the control group. Once the cells reached desired cell confluence for differentiation, the medium was switched from growth medium to either adipogenic or osteogenic differentiation medium (StemPro Differentiation Kit, Thermo Fisher Scientific, Schwerte, Germany), which contained varying concentrations of H_2_O_2_ to induce stem cell differentiation. The differentiation medium was refreshed every 3 d. Adipogenic and osteogenic differentiation were assessed after 14 d and 21 d, respectively.

For assessment, cells were first rinsed with PBS and fixed with 3.7 wt.% paraformaldehyde (Sigma–Aldrich, Hamburg, Germany) for 15 min. After further rinsing with distilled water, cells were stained with 0.36 wt.% Oil Red O (Sigma–Aldrich, Hamburg, Germany) in 60 vol% isopropanol at room temperature for 15 min to visualize lipids in adipocytes. Alternatively, cells were stained with 40 mM Alizarin Red S (Sigma–Aldrich, Hamburg, Germany) in distilled water for 20 min to detect calcium deposits. Images were captured using an Axiovert 40C microscope (Carl Zeiss, Jena, Germany). Lipid and mineralization areas were quantified using ImageJ software.

Flow cytometry analysis was performed to determine the effect of PVA‐GA hydrogel on the typical phenotypic surface markers of hADSCs. After 14 d of co‐culture with the hydrogel, hADSCs were stained with CD90‐FITC, CD105‐FITC, CD271‐APC, and CD73‐APC antibodies (Miltenyi Biotec, Bergisch Gladbach, Germany). Corresponding isotype antibodies were used as controls. Stained cells were then analyzed using a MACSQuant flow cytometer (Miltenyi Biotec, Bergisch Gladbach, Germany), and the results were processed with FlowJo software (Tree Star Inc., Ashland, OR, USA).

### VEGF Secretion

The hADSCs (passage 6) were seeded into 24‐well TCP at a density of 1.0 × 10^4^ cells per well. The hydrogel (1 wt.% GA) was applied at a dose of 4 µL per well, with pure PVA and GA added in equivalent amounts to match the hydrogel composition. For the H_2_O_2_‐treated groups, the cells were exposed to 0.25 mM H_2_O_2_ (prepared in FBS‐free DMEM) for 2 h. After treatment, the cells were cultured in low FBS (2%) DMEM medium. After 7 days of cultivation, the conditioned medium was collected. The concentration of VEGF‐A was measured using the Human VEGF‐A ELISA Kit (Thermo Fisher Scientific, Schwerte, Germany), with fluorescence intensity assessed via a microplate reader (Infinite 200 Pro, Tecan Group Ltd., Männedorf, Switzerland).

### Wound Healing Assay

The wound healing assay was performed by seeding HUVECs into the Culture‐Insert 3 Well in µ‐Dish (ibid, Gräfelfing, Germany) following the provided protocol. After cell attachment, cell‐free gaps were created to visualize cell migration. HUVECs were cultured with different conditioned medium, and images were captured at 4 and 8 h using an Axiovert 40C microscope (Carl Zeiss, Jena, Germany). The scratch areas were measured using ImageJ, which automatically detects the edge positions. The wound closure percentage was calculated by assessing the change in normalized area relative to the original open area, using the formula: *Wound Closure % = [(A_t = 0_ – A_t__= Δh_) / A_t = 0_] × 100*. In this formula, A_t = 0_ represents the area at the initial time point, while A_t__=__Δh_ denotes the area after a specified incubation period (h).

### Tube Formation Assay

The 24‐well plate was pre‐coated with Geltrex LDEV‐Free Reduced Growth Factor Basement Membrane Matrix (Thermo Fisher Scientific, Schwerte, Germany). HUVECs were seeded at a density of 5.0 × 10^4^ cells well^−1^, and were cultured with different conditioned media for 12 h. Following this incubation, the cells were stained with Calcein AM (Thermo Fisher Scientific, Schwerte, Germany), and images were captured using a confocal laser scanning microscope (LSM780, Carl Zeiss, Jena, Germany). Image analysis was performed using a program developed for the ImageJ software. This plugin is an extension of the “Angiogenesis Analyzer” for ImageJ,^[^ ^59^ ^]^ written in the macro language of ImageJ.

### Cell Sedimentation

To detect the sedimentation of cells during the gelation process after mixing with hydrogel, first, hADSCs were stained using the CellTrace CFSE Cell Proliferation Kit (Thermo Fisher Scientific, Schwerte, Germany) according to the provided protocol. The stained cells were then incorporated into hydrogels with different GA concentrations (0.5 wt.% and 1 wt.%). Images were captured at 0 and 30 min for the hydrogel (GA 0.5 wt.%) and at 0 and 10 min for the hydrogel (GA 1 wt.%), corresponding to their respective gelation times. A confocal laser scanning microscope (LSM780, Carl Zeiss, Jena, Germany) was used for imaging.

### Statistical Analysis

The number of replications for each experiment was indicated in the figure legends. Data were presented as mean value ± standard deviation. Statistical analysis was performed using one‐way or two‐way analysis of variance (ANOVA) as indicated for each experiment, followed by Tukey's test using the GraphPad Prism software (Version 10.0, San Diego, USA). A *p*‐value less than 0.05 was considered statistically significant.

## Conflict of Interest

The authors declare no conflict of interest.

## Supporting information



Supporting Information

## Data Availability

The data that support the findings of this study are available from the corresponding author upon reasonable request.

## References

[1] M. R. Kouchakian , N. Baghban , S. F. Moniri , M. Baghban , S. Bakhshalizadeh , V. Najafzadeh , Z. Safaei , S. Izanlou , A. Khoradmehr , I. Nabipour , R. Shirazi , A. Tamadon , Stem Cells Int. 2021, 2021, 1634782. 34745268 10.1155/2021/1634782 PMC8566082

[2] U. Galderisi , G. Peluso , G. Di Bernardo , Stem Cell Rev. Rep. 2022, 18, 23. 34398443 10.1007/s12015-021-10231-w PMC8365566

[3] D. Jovic , Y. Yu , D. Wang , K. Wang , H. Li , F. Xu , C. Liu , J. Liu , Y. Luo , Stem Cell Rev. Rep. 2022, 18, 1525. 35344199 10.1007/s12015-022-10369-1 PMC8958818

[4] D. M. Hoang , P. T. Pham , T. Q. Bach , A. T. L. Ngo , Q. T. Nguyen , T. T. K. Phan , G. H. Nguyen , P. T. T. Le , V. T. Hoang , N. R. Forsyth , M. Heke , L. T. Nguyen , Signal Transduction Targeted Ther. 2022, 7, 272. 10.1038/s41392-022-01134-4 PMC9357075 35933430

[5] P. S. Liu , Front. Cell. Infect. Microbiol. 2023, 13, 1137947. 37091673 10.3389/fcimb.2023.1137947 PMC10117668

[6] W. Wang , X. Xu , Z. Li , A. Lendlein , N. Ma , Clin. Hemorheol. Microcirc. 2014, 58, 19. 25227201 10.3233/CH-141883

[7] W. Wang , Z. Deng , X. Xu , Z. Li , F. Jung , N. Ma , A. Lendlein , Curr. Pharm. Des. 2017, 23, 3814. 28641542 10.2174/1381612823666170622110654

[8] W. Li , N. Ma , L.‐L. Ong , C. Nesselmann , C. Klopsch , Y. Ladilov , D. Furlani , C. Piechaczek , J. M. Moebius , K. Lützow , A. Lendlein , C. Stamm , R.‐K. Li , G. Steinhoff , Stem Cells 2007, 25, 2118. 17478584 10.1634/stemcells.2006-0771

[9] W. Huang , L. J. Hickson , A. Eirin , J. L. Kirkland , L. O. Lerman , Nat. Rev. Nephrol. 2022, 18, 611. 35922662 10.1038/s41581-022-00601-z PMC9362342

[10] C. A. Juan , J. M. Pérez de la Lastra , F. J. Plou , E. Pérez‐Lebeña , Int. J. Mol. Sci. 2021, 22, 4642. 33924958 10.3390/ijms22094642 PMC8125527

[11] M. Schieber , N. S. Chandel , Curr. Biol. 2014, 24, 4642. 10.1016/j.cub.2014.03.034 PMC4055301 24845678

[12] Z. J. Deng , W. Wang , X. Xu , Y. Nie , Y. Liu , O. E. C. Gould , N. Ma , A. Lendlein , ACS Appl. Mater. Interfaces 2021, 13, 10748. 33594879 10.1021/acsami.0c22565

[13] F. Atashi , A. Modarressi , M. S. Pepper , Stem Cells Dev. 2015, 24, 1150. 25603196 10.1089/scd.2014.0484 PMC4424969

[14] T. O. Waheed , Int. J. Mol. Sci. 2022, 23, 13435.36362223

[15] R. A. Denu , P. Hematti , Oxid. Med. Cell. Longev. 2016, 2016, 2989076. 27413419 10.1155/2016/2989076 PMC4928004

[16] Q. C. Pereira , T. W. D. Santos , I. M. Fortunato , M. L. Ribeiro , Int. J. Mol. Sci. 2023, 24, 5508.36982583

[17] J. H. Lee , J. Park , D. W. Shin , Molecules 2022, 27, 4351.35889225

[18] E. Csekes , L. Racková , Int. J. Mol. Sci. 2021, 22, 12641. 34884444 10.3390/ijms222312641 PMC8657738

[19] J. Gao , J. Hu , D. Hu , X. Yang , Nat. Prod. Commun. 2019, 14.

[20] S. Verma , A. Singh , A. Mishra , Environ. Toxicol. Pharmacol. 2013, 35, 473. 23501608 10.1016/j.etap.2013.02.011

[21] B. Badhani , N. Sharma , R. Kakkar , RSC Adv. 2015, 5, 27540.

[22] H. Z. Shan , L. Geng , X. Jiang , M. Song , J. Wang , Z. Liu , X. Zhuo , Z. Wu , J. Hu , Z. Ji , Protein Cell 2022, 13, 532. 34542813 10.1007/s13238-021-00872-5 PMC9226226

[23] J. Tang , P. Zhang , Y. Liu , D. Hou , Y. Chen , L. Cheng , Y. Xue , J. Liu , Biomaterials 2025, 314, 122880. 39383777 10.1016/j.biomaterials.2024.122880

[24] Y. X. Pang , L. Guan , Y. Zhu , R. Niu , S. Zhu , Q. Lin , Front. Bioeng. Biotechnol. 2023, 11, 1162202. 37334266 10.3389/fbioe.2023.1162202 PMC10273101

[25] W. Weian , Y. Yunxin , W. Ziyan , J. Qianzhou , G. Lvhua , Biomater. Sci. 2024, 12, 1405. 38372381 10.1039/d3bm01925j

[26] G. C. Yen , P. D. Duh , H. L. Tsai , Food Chem. 2002, 79, 307.

[27] E. M. Euti , A. Wolfel , M. L. Picchio , M. R. Romero , M. Martinelli , R. J. Minari , C. I. Alvarez Igarzabal , Macromol. Rapid Commun. 2019, 40, 1900217. 10.1002/marc.201900217 31535770

[28] A. Carnicero , A. Gonzalez , S. D. Dalosto , M. C. G. Passeggi Jr. , R. J. Minari , C. I. Alvarez Igarzabal , M. Martinelli , M. L. Picchio , ACS Biomater. Sci. Eng. 2022, 8, 5027. 36318285 10.1021/acsbiomaterials.2c00935

[29] A. Wolfel , E. M. Euti , M. L. Picchio , M. R. Romero , V. M. Galvan Josa , M. Martinelli , R. J. Minari , C. I. Alvarez Igarzabal , Polym. Chem. 2020, 11, 7185.

[30] G. C. Luque , M. L. Picchio , A. P. S. Martins , A. Dominguez‐Alfaro , L. C. Tomé , D. Mecerreyes , R. J. Minari , Macromol. Biosci. 2020, 20, 2000119. 10.1002/mabi.202000119 32597002

[31] V. R. Feig , H. Tran , M. Lee , Z. Bao , Nat. Commun. 2018, 9, 2740. 30013027 10.1038/s41467-018-05222-4 PMC6048132

[32] O. Chaudhuri , L. Gu , D. Klumpers , M. Darnell , S. A. Bencherif , J. C. Weaver , N. Huebsch , H.‐P. Lee , E. Lippens , G. N. Duda , D. J. Mooney , Nat. Mater. 2016, 15, 326. 26618884 10.1038/nmat4489 PMC4767627

[33] Y. He , K. Liu , C. Zhang , S. Guo , R. Chang , F. Guan , M. Yao , Biomater. Sci. 2022, 10, 5620. 35989642 10.1039/d2bm00891b

[34] Z. Deng , W. Wang , X. Xu , O. E. C. Gould , K. Kratz , N. Ma , A. Lendlein , Proc. Natl. Acad. Sci. USA 2020, 117, 1895. 31932451 10.1073/pnas.1910668117 PMC6995006

[35] M. Perez‐Araluce , T. Jüngst , C. Sanmartin , F. Prosper , D. Plano , M. M. Mazo , Biomimetics 2024, 9, 23. 38248597 10.3390/biomimetics9010023 PMC10813727

[36] Y. Zhong , X. Li , F. Tan , Y. Fei , X. Zhao , J. Xu , B. Fan , Front. Chem. 2024, 12, 123.

[37] L. H. Russell , E. Mazzio , R. B. Badisa , Z.‐P. Zhu , M. Agharahimi , E. T. Oriaku , C. B. Goodman , Anticancer Res. 2012, 32, 1595. 22593437 PMC3356927

[38] P. Davalli , T. Mitic , A. Caporali , A. Lauriola , D. D'Arca , Oxid. Med. Cell. Longev. 2016. 10.1155/2016/3565127 PMC4877482 27247702

[39] G. W. Ye , Z. Xie , H. Zeng , P. Wang , J. Li , G. Zheng , S. Wang , Q. Cao , M. Li , W. Liu , S. Cen , Z. Li , Y. Wu , Z. Ye , H. Shen , Cell Death Dis. 2020, 11, 775. 32943613 10.1038/s41419-020-02993-x PMC7498590

[40] N. Kumar , N. Goel , Biotechnol Rep (Amst) 2019, 24, 00370. 10.1016/j.btre.2019.e00370 PMC6734135 31516850

[41] S. Kumari , A. K. Badana , M. Mohan G , G. Shailender , R. Malla , Biomark Insights 2018, 13, 1177271918755391. 29449774 10.1177/1177271918755391 PMC5808965

[42] J. S. Bhatti , G. K. Bhatti , P. H. Reddy , Biochim. Biophys. Acta‐Mol. Basis Dis. 2017, 1863, 1066. 27836629 10.1016/j.bbadis.2016.11.010 PMC5423868

[43] P. Kowalczyk , D. Sulejczak , P. Kleczkowska , I. Bukowska‐Osko , M. Kucia , M. Popiel , E. Wietrak , K. Kramkowski , K. Wrzosek , K. Kaczynska , Int. J. Mol. Sci. 2021, 22, 13384. 34948180 10.3390/ijms222413384 PMC8707347

[44] Y. Zong , H. Li , P. Liao , L. Chen , Y. Pan , Y. Zheng , C. Zhang , D. Liu , M. Zheng , J. Gao , Signal Transduct. Target. Ther. 2024, 9, 124. 38744846 10.1038/s41392-024-01839-8 PMC11094169

[45] A. Picerno , A. Stasi , R. Franzin , C. Curci , I. di Bari , L. Gesualdo , F. Sallustio , World J. Stem Cells 2021, 13, 1714. 34909119 10.4252/wjsc.v13.i11.1714 PMC8641024

[46] J. C. Estrada , Y. Torres , A. Benguria , A. Dopazo , E. Roche , L. Carrera‐Quintanar , R. A. Perez , J. A. Enriquez , R. Torres , J. C. Ramirez , E. Samper , A. Bernad , Cell Death Dis. 2013, 4, 691. 10.1038/cddis.2013.211 PMC3702285 23807220

[47] W. Wagner , P. Horn , M. Castoldi , A. Diehlmann , S. Bork , R. Saffrich , V. Benes , J. Blake , S. Pfister , V. Eckstein , A. D. Ho , PLoS One 2008, 3, 2213. 10.1371/journal.pone.0002213 PMC2374903 18493317

[48] E. Iakovou , M. Kourti , Front. Aging Neurosci. 2022, 14, 827900. 35769600 10.3389/fnagi.2022.827900 PMC9234325

[49] X. K. Zhou , Y. Hong , H. Zhang , X. Li , Front. Cell Dev. Biol. 2020, 8, 00364. 10.3389/fcell.2020.00364 PMC7283395 32582691

[50] S. Petersen , G. Saretzki , T. von Zglinicki , Exp. Cell Res. 1998, 239, 152. 9511733 10.1006/excr.1997.3893

[51] C. López‐Otín , M. A. Blasco , L. Partridge , M. Serrano , G. Kroemer , Cell 2013, 153, 1194. 23746838 10.1016/j.cell.2013.05.039 PMC3836174

[52] H. Martini , J. F. Passos , FEBS J. 2023, 290, 1186. 35048548 10.1111/febs.16361 PMC9296701

[53] R. Salama , M. Sadaie , M. Hoare , M. Narita , Genes Dev. 2014, 28, 99. 24449267 10.1101/gad.235184.113 PMC3909793

[54] M. Z. Ratajczak , M. Kucia , T. Jadczyk , N. J. Greco , W. Wojakowski , M. Tendera , J. Ratajczak , Leukemia 2012, 26, 1166. 22182853 10.1038/leu.2011.389

[55] Y. Jin , S. Li , Q. Yu , T. Chen , D. Liu , MedComm 2020, 4, 291. 10.1002/mco2.291 PMC10276889 37337579

[56] D. I. R. Holmes , I. Zachary , Genome Biol. 2005, 6, 209. 15693956 10.1186/gb-2005-6-2-209 PMC551528

[57] L. Guo , J. Du , D.‐F. Yuan , Y. Zhang , S. Zhang , H.‐C. Zhang , J.‐W. Mi , Y.‐L. Ning , M.‐J. Chen , D.‐L. Wen , J.‐H. Sun , D. Liu , L. Zeng , A. Zhang , J. Jiang , H. Huang , Stem Cell Res. Ther. 2020, 11, 434. 33032649 10.1186/s13287-020-01910-5 PMC7545926

[58] A. Takahashi , H. Nakajima , A. Kubota , S. Watanabe , A. Matsumine , Cells 2023, 12, 1470. 37296591 10.3390/cells12111470 PMC10252677

[59] G. Carpentier , S. Berndt , S. Ferratge , W. Rasband , M. Cuendet , G. Uzan , P. Albanese , Sci. Rep. 2020, 10, 11568. 32665552 10.1038/s41598-020-67289-8 PMC7360583

