# Supplementary material for: Antioxidant and Anti‐Senescence Polyvinyl Alcohol‐Gallic Acid Supramolecular Hydrogels for Stem Cell Culture
Source: Adv Healthc Mater. 2025 Apr 17;14(17):2402882. doi: 10.1002/adhm.202402882 (PMC12232133; doi:10.1002/adhm.202402882)

**Supporting Information:**

**Antioxidant and Anti-senescence Polyvinyl Alcohol-Gallic Acid Supramolecular Hydrogels for Stem Cell Culture**

Yiduo Zhou^1, 2, 3,^ **^#^**, Matías L. Picchio ^4, 5, 6, 7^ **^#^**, Yan Nie ^2, 3^, Lei Wang ^1, 2, 3^, Oihane Sanz ^4^, Yue Liu ^2, 3^, Xun Xu ^2, 3^, Lukas Prantl ^8^, Oliver Felthaus ^8^, Weiwei Wang ^2, 3,^ *****, Marcelo Calderón ^4, 5,^ *****, Nan Ma ^1, 2, 3,^ *****

**Affiliations:**

^1^ Institute of Chemistry and Biochemistry, Free University of Berlin, 14195, Berlin, Germany

^2^ Institute of Active Polymers, Helmholtz-Zentrum Hereon, 14513, Teltow, Germany

^3^ Institute of Functional Materials for Sustainability, Helmholtz-Zentrum Hereon, 14513 Teltow, Germany

^4^ POLYMAT, Applied Chemistry Department, Faculty of Chemistry, University of the Basque Country UPV/EHU, Paseo Manuel de Lardizábal 3, Donostia-San Sebastián, 20018, Spain

^5^ IKERBASQUE, Basque Foundation for Science, Plaza Euskadi 5, Bilbao, 48009, Spain

^6^ Universidad Tecnológica Nacional, Facultad Regional Villa María, Av. Universidad 450 (5900) Villa María, Córdoba, Argentina

^7^ Consejo Nacional de Investigaciones Científicas y Técnicas (CONICET), Godoy Cruz 2290, CABA, Buenos Aires, Argentina

^8^ Department of Plastic, Hand and Reconstructive Surgery, University Hospital Regensburg, Franz-Josef-Strauß-Allee 11, 93053, Regensburg, Germany

# These authors contributed equally to this work.

*Correspondence to: weiwei.wang@hereon.de, marcelo.calderonc@ehu.eus, nan.ma@fu-berlin.de


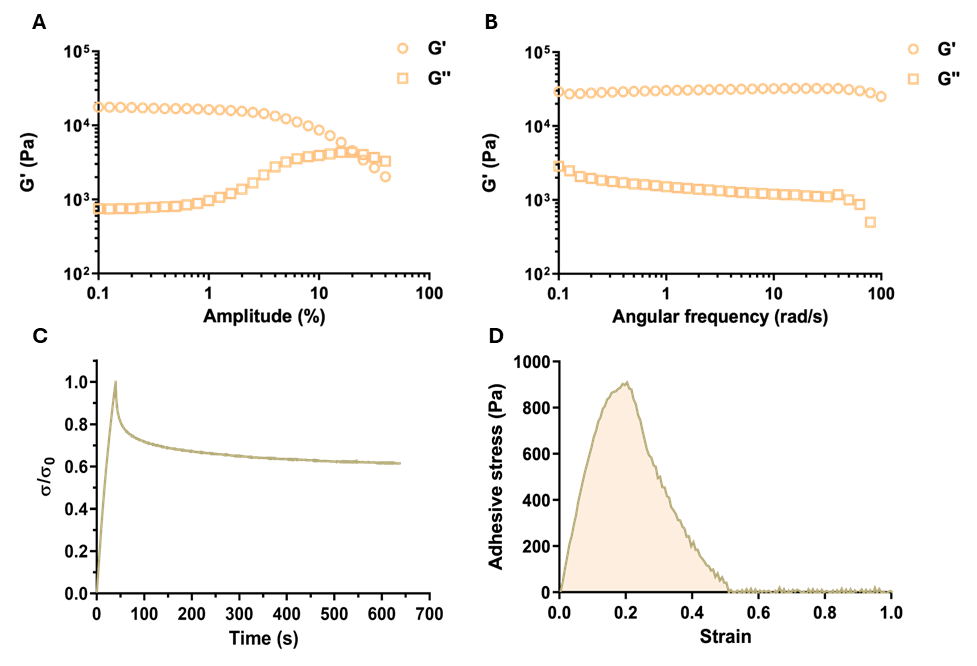


**Figure S1**. Characterization of PVA-GA hydrogel with 1 wt.% GA. A) Amplitude and B) angular frequency sweep according to small amplitude oscillatory shear, C) stress-relaxation curve (σ_0_ = maximum stress), and D) adhesive stress vs strain curve.


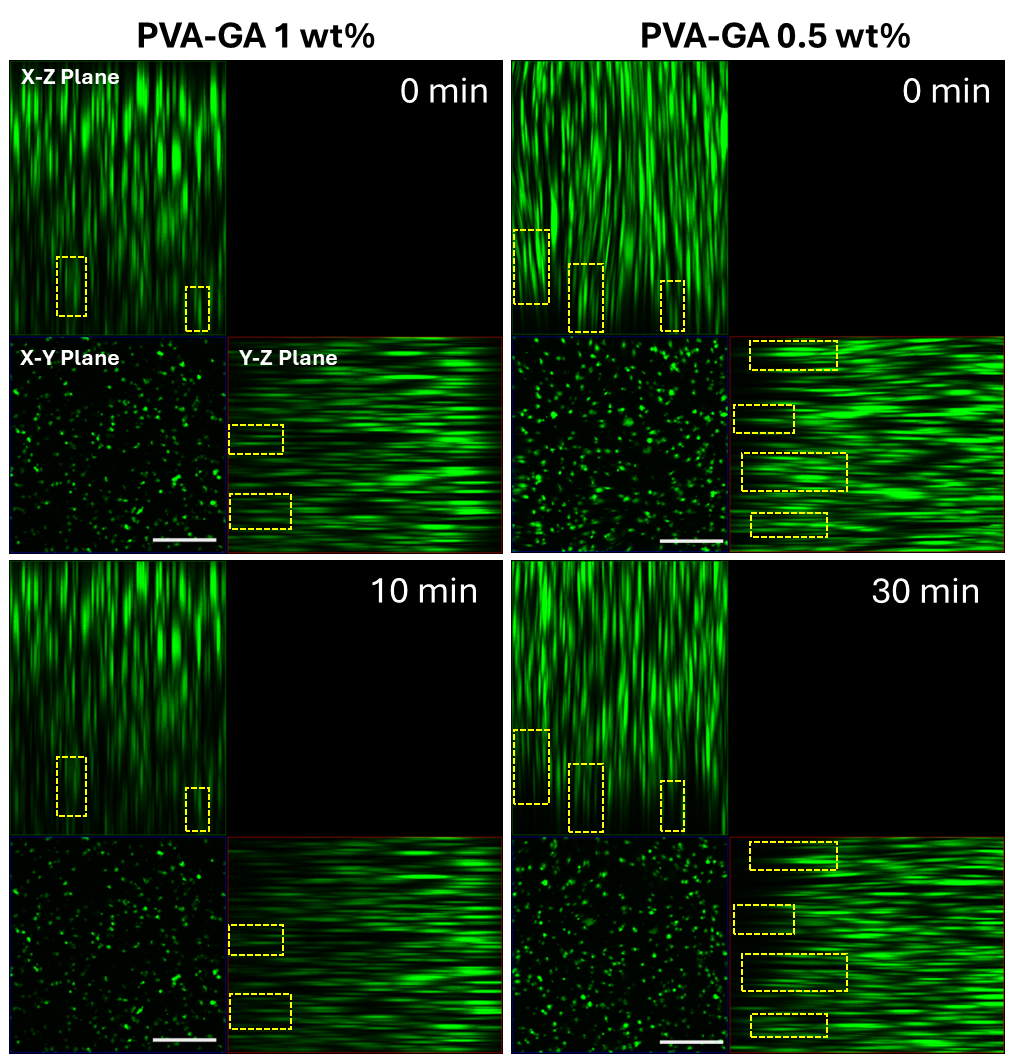


**Figure S2**. Cell sedimentation in PVA-GA hydrogels containing 1 wt.% and 0.5 wt.% GA. hADSCs were stained with CellTrace™ CFSE and mixed with the PVA-GA solution. The mixture was incubated at 4°C to facilitate gelation. At the indicated time points, cells were visualized using a confocal laser scanning microscope. The z-axis scanning range was 650 µm, and images are presented as maximum intensity projections from different planes. Yellow rectangle frames indicated the cell sedimentation during gelation process. Compared to the hydrogel with 0.5 wt.% GA, the hydrogel with 1 wt.% GA gelled rapidly at 4°C, resulting in swifter cell immobilization, as evidenced by negligible horizontal movement and slight vertical sedimentation of the cell. (scale bar = 500 µm)


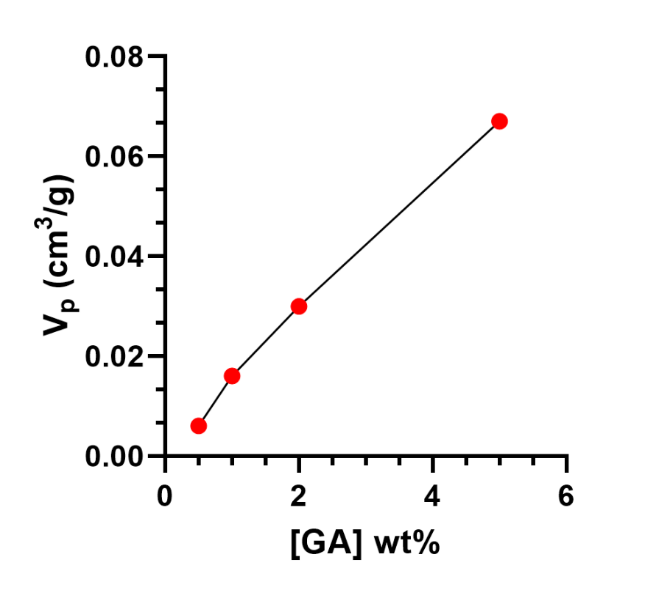


**Figure S3.** Pore volume (V_p_) *vs.* GA concentration of the hydrogels obtained by N2 physisorption at a relative pressure, P/P_0_ = 0.99.


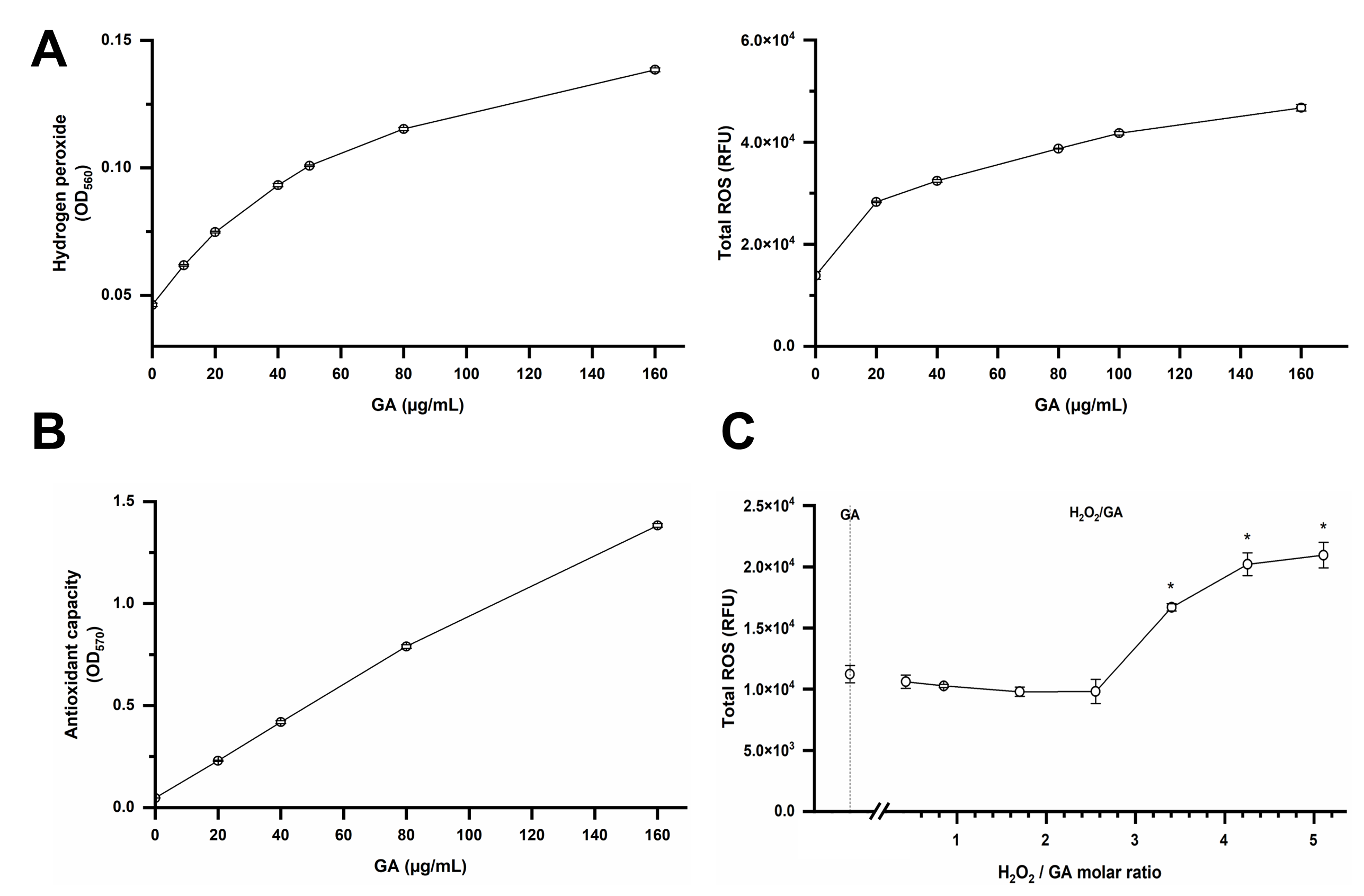


**Figure S4.** Autoxidation and Antioxidation of GA. A) Levels of H_2_O_2_ and total ROS in aqueous solutions of pure GA at varying concentrations, quantified after 24-hour incubation at 37°C (n ≥ 3). B) Antioxidant capacity of pure GA at varying concentrations (n = 3). C) Antioxidant capacity of GA under varying ROS levels (expressed as the molar ratio of H_2_O_2_ to GA) (n = 3, **p* < 0.05, one-way ANOVA with Tukey’s multiple comparisons test).

**
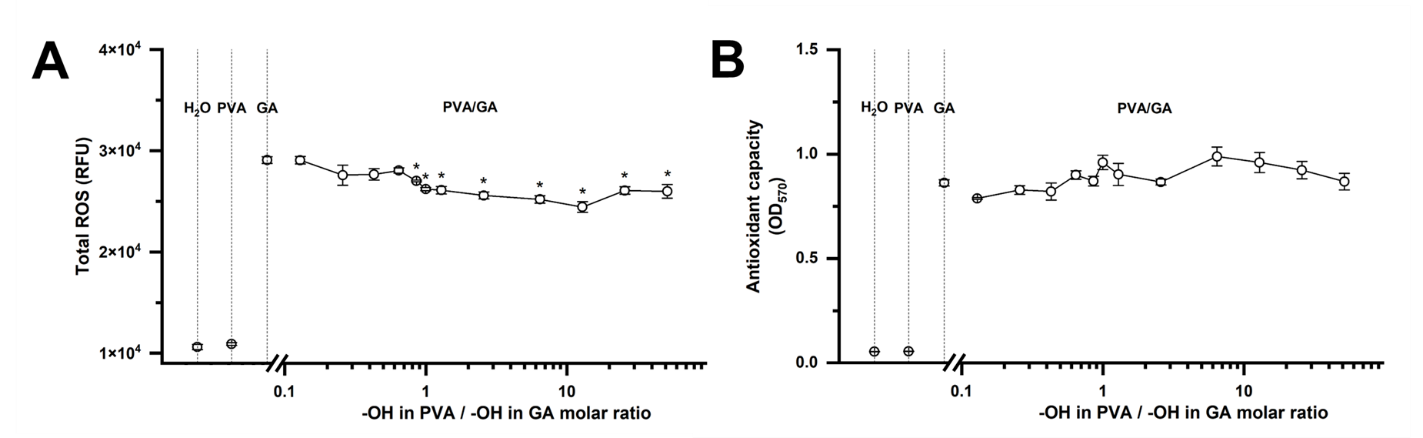
**

**Figure S5.** Autoxidation and Antioxidation of PVA/GA mixture. A) Total ROS levels in aqueous solutions containing PVA and GA at varying PVA/GA ratios, measured after 24-hour incubation at 37°C. B) Antioxidant capacity of GA in the presence of PVA. The PVA/GA ratio is expressed as the molar ratio of hydroxyl groups in PVA to phenolic hydroxyl groups in GA. Pure water, PVA and GA were included as controls (n = 3, **p* < 0.05 compared to pure GA, one-way ANOVA with Tukey’s multiple comparisons test).


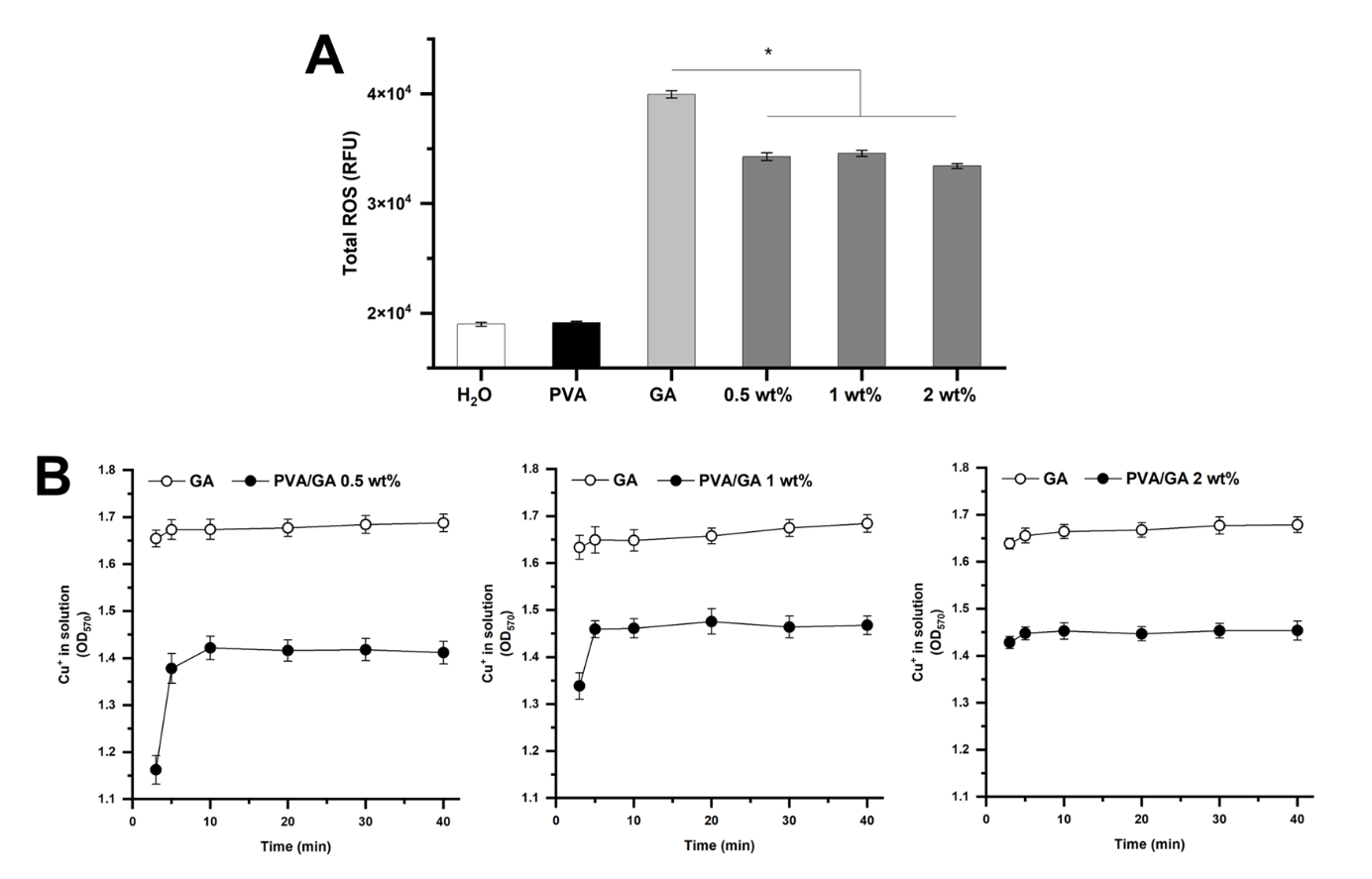


**Figure S6.** Autoxidation and Antioxidation of PVA-GA hydrogel. A) Total ROS levels in aqueous solutions containing PVA-GA hydrogels with varying GA percentages, assessed after 24-hour incubation at 37°C. Pure water, PVA and GA were included as controls (n = 4, **p* < 0.05, one-way ANOVA with Tukey’s multiple comparisons test). B) Antioxidant capacity of PVA-GA hydrogels containing 0.5, 1, and 2 wt.% GA, compared to equivalent amounts of pure GA. The graphs show detectable Cu^+^ levels in the solution as a function of incubation time (n ≥ 3).


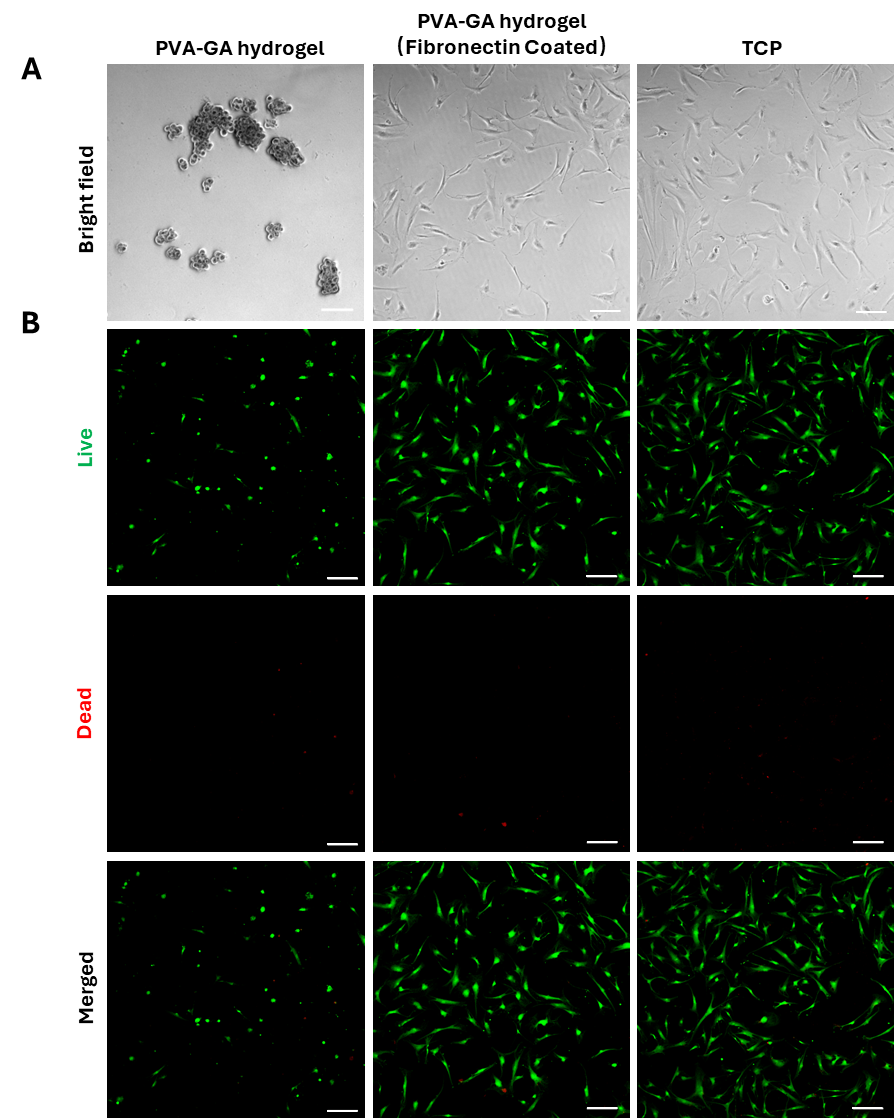


**Figure S7**. hADSCs cultured directly on PVA-GA hydrogel and fibronectin-coated PVA-GA hydrogel. A) Morphology and B) viability of hADSCs on PVA-GA and fibronectin coated PVA-GA hydrogel, 24 hours post-cell seeding. hADSCs culture on TCP served as control for comparison (scale bar = 50 μm).

**
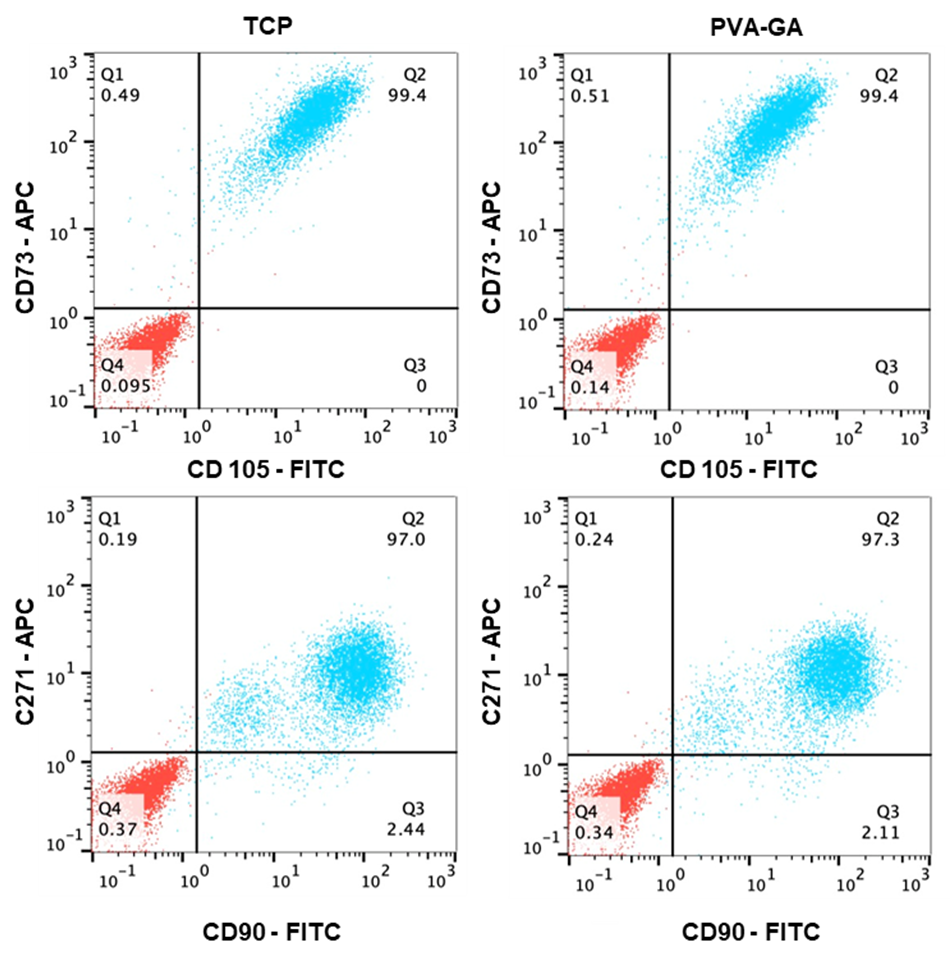
**

**Figure S8.** Flow cytometric analysis of phenotypic markers of hADSCs cultured with and without hydrogel.

**Table S1.** **Cell surface marker phenotypes of hADSCs**


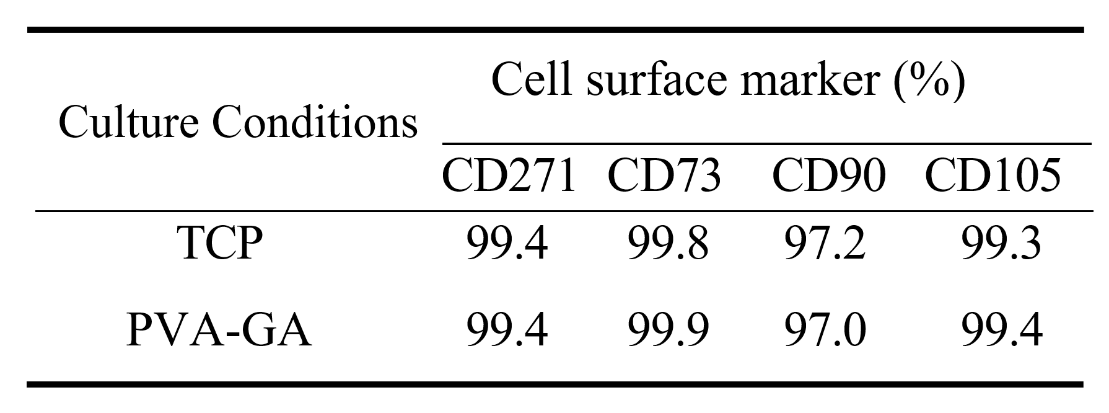

Supplement: Supplementary file 1 — Supporting Information [file ADHM-14-0-s001.docx]
